# Carbon nanotubes targeted to the tumor microenvironment inhibit metastasis in a preclinical model of melanoma

**DOI:** 10.1016/j.bioactmat.2023.12.013

**Published:** 2023-12-28

**Authors:** Lorena García-Hevia, Rym Soltani, Jesús González, Olivier Chaloin, Cécilia Ménard-Moyon, Alberto Bianco, Mónica L. Fanarraga

**Affiliations:** a The Nanomedicine Group, Universidad de Cantabria-IDIVAL, Avda Herrera Oria s/n, 39011, Santander, Spain; b CNRS, Immunology, Immunopathology and Therapeutic Chemistry, UPR 3572, University of Strasbourg, ISIS, 67000, Strasbourg, France

**Keywords:** Carbon nanomaterials, Nanofilaments, Vascularization, Peptide conjugates, Angiogenesis

## Abstract

Despite notable progress in cancer therapy, metastatic diseases continue to be the primary cause of cancer-related mortality. Multi-walled carbon nanotubes (MWCNTs) can enter tissues and cells and interfere with the dynamics of the cytoskeletal nanofilaments biomimetically. This endows them with intrinsic anti-tumoral effects comparable to those of microtubule-binding chemotherapies such as Taxol®.

In this study, our focus was on exploring the potential of oxidized MWCNTs in selectively targeting the vascular endothelial growth factor receptor (VEGFR). Our objective was to evaluate their effectiveness in inhibiting metastatic growth by inducing anti-proliferative, anti-migratory, and cytotoxic effects on both cancer and tumor microenvironment cells. Our findings demonstrated a significant reduction of over 80 % in malignant melanoma lung metastases and a substantial enhancement in overall animal welfare following intravenous administration of the targeted biodegradable MWCNTs. Furthermore, the combination of these nanomaterials with the conventional chemotherapy agent Taxol® yielded a remarkable 90 % increase in the antimetastatic effect. These results highlight the promising potential of this combined therapeutic approach against metastatic disease and are of paramount importance as metastasis is responsible for nearly 60,000 deaths each year.

## Highlights

• Carbon nanotubes mimic cytoskeletal filaments, showing intrinsic antitumoral effects like tubulin-binding chemotherapies in cells/tissues.
• Targeting VEGF receptor with CNTs inhibits metastatic growth via anti-proliferative, anti-migratory, and cytotoxic.
• Intravenous treatment with targeted biodegradable nanotubes reduced lung metastases by over 80% and significantly improved survival.
• Combining the targeted nanotubes with Taxol®, a traditional chemotherapy drug, increased the anti-metastatic effect by 90%.
• This study highlights the potential of using tumor microenvironment-targeted carbon nanotubes to combat the metastasis of malignant tumors.

## Graphical abstract

Carbon nanotubes (CNTs) disrupt the dynamics of the cell cytoskeleton, leading to the inhibition of cell proliferation and migration, ultimately triggering antitumor activity. Here, we effectively direct biodegradable CNTs to lung metastases by employing a neovasculature-binding peptide, achieving a 90 % reduction in metastases with just three doses of CNT-conjugates. Our study provides evidence that these CNT-conjugates synergistically enhance the efficacy of Taxol® (paclitaxel) in preclinical models of metastatic malignant melanoma.

**Figure:**
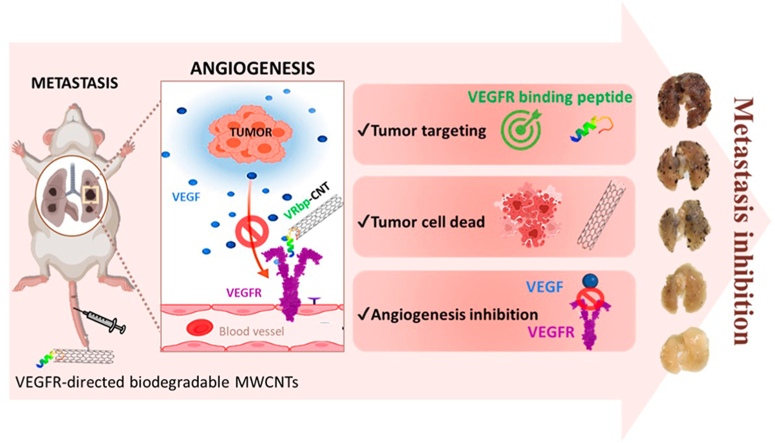

## 1 Introduction

Despite recent therapeutic advances in cancer treatment, metastatic diseases remain the leading cause of cancer death. Metastases cannot be localized in time since they emerge in a disseminated form in various tissues, making surgery and radiotherapy impractical for treatment and sometimes even promoting tumor metastasis [1]. Consequently, cytotoxic chemotherapy, which entails the use of drugs that selectively target rapidly dividing cells, has traditionally constituted a primary approach for managing metastasis across various cancer types. While effective in targeting fast-growing cancer cells, cytotoxic chemotherapy is often associated with significant side effects, such as damage to healthy tissue and impaired immune function. In recent years, however, the treatment landscape for metastatic cancer has been transformed by the advent of targeted therapies and immunotherapies. In particular, targeted therapies are designed to inhibit specific molecules or pathways involved in tumor growth and survival, offering potentially more precise and less toxic treatment modalities. These therapies target specific proteins that play a crucial role in the growth, division, and spread of cancer cells, providing a more effective approach to treatment.

**Fig. 1:**
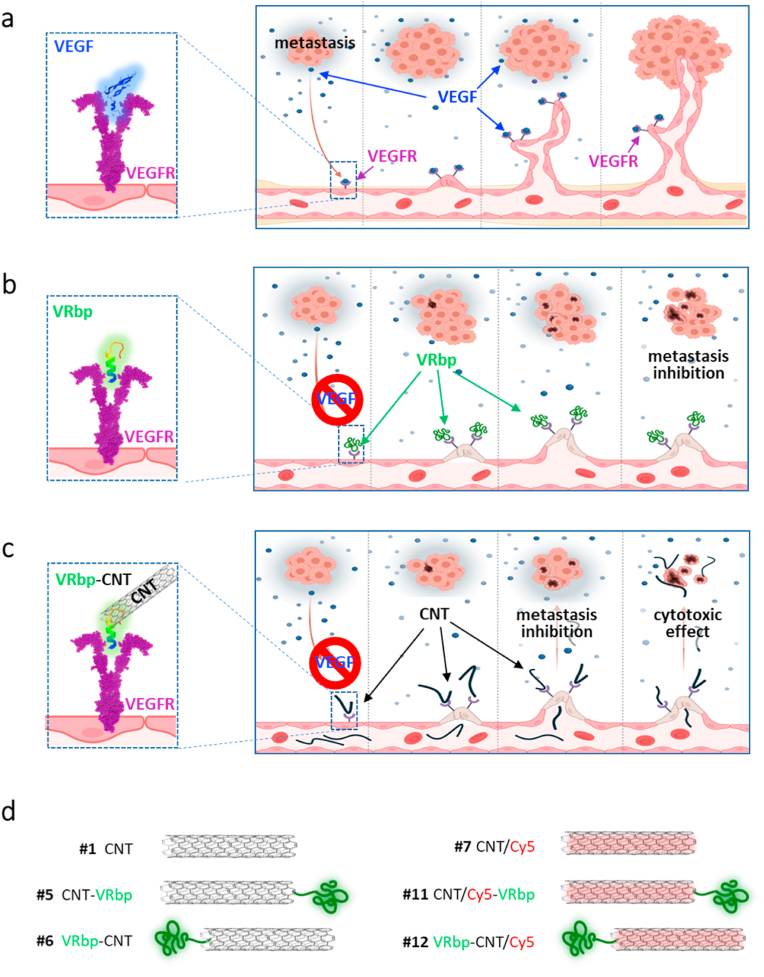
**Diagram of the VEGFR targeting hypothesis.** (a) Interaction between VEGF in blue and its receptor VEGFR in purple, highlighting its influence on metastatic angiogenesis. (b) Interaction of the VEGFR binding-peptide (VRbp) (green) with the receptor, targeting the metastatic tumor microenvironment and resulting in metastasis inhibition. (c) Anticipated outcome of VRbp-functionalized CNT targeting the metastatic tumoral neovasculature, leading to metastasis inhibition and inducing cytotoxic effects on cells.

Since migrating cancer cells produce high levels of vascular endothelial growth factor (VEGF), a secreted angiogenic mitogen that promotes angiogenesis and tumor vascularization, vascular endothelial growth factor receptor (VEGFR) inhibitors are a particularly promising class of targeted therapy [[2], [3], [4], [5]]. Various strategies to disrupt the pro-angiogenic effects of VEGF and inhibit tumor vascularization have been explored. One approach involves neutralizing VEGF or its receptor using antibodies such as bevacizumab or peptides directed against VEGF or its receptor [6]. By targeting the VEGFR signaling pathway, these molecules effectively inhibit angiogenesis, the formation of new blood vessels crucial for tumor growth and metastasis [[7], [8], [9], [10]]. Through the blockade of this process, VEGFR inhibitors restrict the blood supply to tumors, thereby limiting their growth (Fig. 1a–b). Unfortunately, like many targeted therapies, inhibitors of the VEGF pathway produce only transient responses to treatment, due to the emergence of resistant tumor cell subclones [[11], [12], [13]].

Apart from establishing a novel blood vascular network, metastatic cancers necessitate the development of a favorable tumor microenvironment that plays a critical role in cancer progression and treatment response. It comprises a complex network of cells, blood vessels, extracellular matrix, and signaling molecules that surround and interact with cancer cells. This diverse and dynamic milieu influences tumor growth, invasion, metastasis, and immune response. Therefore, targeting these supportive cells could be a promising approach to complement existing treatment options, given their low genetic variability and limited resistance mechanisms compared to cancer cells.

Melanoma, a prevalent form of skin cancer, possesses the potential to rapidly spread and metastasize throughout the body, leading to severe and potentially fatal outcomes if not detected early and surgically removed. The incidence of melanoma has been consistently increasing in Western populations, with the total number of cases doubling over the past two decades [14]. Despite prognosis being affected by factors like metastatic location and quantity, metastatic melanoma typically has a 10-year survival rate of less than 10 % [15]. Hence, there is a pressing need for innovative approaches to address this challenge.

Nanomedicine offers solutions for treating diseases where conventional therapies often fail. It can also improve the effectiveness of chemotherapy [16]. Numerous intelligent nanomaterials have been developed with the specific purpose of delivering chemotherapy drugs to tumors, aiming to optimize the therapeutic efficacy while minimizing the required dosage. However, research indicates that, despite important anti-cancer effects, a negligible proportion, less than 1%, of systemically administered nanosystems successfully reach their intended target site [17,18]. Regrettably, many of these nanosystems are swiftly eliminated from the body, while others are captured by the reticuloendothelial cellular system.

In the field of anti-cancer nanomaterials, some filamentous nanostructures have the potential to disrupt DNA replication and interfere with cellular biomechanics. Notably, carbon nanotubes (CNTs) have emerged as a type of nanomaterial demonstrating inherent anti-tumoral properties. CNTs are highly regarded for their exceptional physicochemical properties, making them attractive for various biotechnological applications [19]. Their substantial specific surface area, coupled with the capacity for diverse chemical functionalization, has led to extensive use in adsorbing or covalently conjugating a wide range of bioactive molecules [[20], [21], [22]]. More interestingly, due to their morphology and surface characteristics, CNTs can infiltrate tissues and cells. This endows them with the innate ability to interfere with the function of intracellular nanofilaments.

Specifically, multi-walled carbon nanotubes (MWCNTs) interact with the microtubules, which are essential tubulin polymers ubiquitous in cell proliferation and migration, triggering antiproliferative [[23], [24], [25]], anti-migratory [26,27], and cytotoxic effects [24,25,[28], [29], [30]]. These two filaments share characteristics, such as tubular structure and specific physical properties like shear stress, bending stiffness, and Young's modulus [31,32]. These resemblances facilitate their interactions with tubulin both *in vitro* [33] and *in vivo* [20,34], resulting in the formation of mixed biosynthetic tubulin polymers exhibiting enhanced stability [23]. In actively dividing cells, MWCNTs prompt notable biomechanical changes, causing mitotic blockage and cell death. Together, these effects contribute to the remarkable inherent anti-tumor properties of MWCNTs observed *in cellulo* and in preclinical models [34,35].

The microtubule-stabilizing properties of MWCNTs are similar to those triggered by paclitaxel (Taxol®), a conventional drug used in cancer treatment [36], so the combination of these two cytoskeletal targeting agents has synergistic actions that can effectively counteract the development of resistance [25,34]. Regrettably, the utilization of CNTs in disease treatment has encountered significant uncertainty, largely attributed to apprehensions about their bio-persistence. This factor has impeded their progress towards clinical applications [[37], [38], [39]]. However, there is now promising headway in the realm of nanomedicine, particularly in the application of oxidized MWCNTs. Preclinical studies have demonstrated that surface oxidation of MWCNTs significantly improves their biocompatibility and biodegradability in living organisms, presenting encouraging potential for *in vivo* applications [35,[40], [41], [42], [43], [44], [45], [46]]. This opens a plethora of new opportunities for the utilization of MWCNTs in the field of nanomedicine [[47], [48], [49]].

Here, we examined the impact of oxidized MWCNTs targeted at the VEGFR on the metastatic progression of malignant melanoma. To achieve this, we directed the nanotubes toward this receptor using a synthetic peptide that binds and interferes with this signaling pathway [50]. The underlying hypothesis for this strategy is to achieve a multiple therapeutic effect. First, by targeting the cells responsible for forming the tumor's neovasculature; second, by inducing tumor cell death; and third, by killing cells in the local microenvironment that support tumor growth, all of which ultimately inhibit the establishment and proliferation of cancer cells (Fig. 1). In our study, we administered biodegradable oxidized MWCNTs intravenously to mice with metastatic melanoma to examine the effect of the targeted nanotubes. In addition, we wanted to investigate the potential adjuvant effect of the combination of VEGFR-targeted oxidized MWCNTs with Taxol® treatment in the inhibition of metastatic growth.

## 2 Materials and Methods

Pristine MWCNTs (9.5 nm diameter, 1.5 μm length) were purchased from Nanocyl (NC3100™, 95%C purity). Cyanine 5 (Cy5) was provided by the group of Dr. Anthony Romieu (Université de Bourgogne, Dijon, France). All reagents were obtained from commercial suppliers and used without further purification. Sulfuric acid, 4 M HCl in 1,4-dioxane, nitric acid, and the dry solvents were purchased from Sigma-Aldrich and used as supplied. 6-maleimide hexanoic acid was purchased from Alfa Aesar. Boc-amino PEG-amine (*O*-(2-aminoethyl)-*O′*-[2-(Boc-amino)ethyl]decaethylene glycol) was obtained from Polypure. Water was purified using a Millipore filter system MilliQ® equipped with a Biopak® filter for pyrogen-free, DNase-free, and RNase-free ultrapure water. MWCNT suspensions were sonicated in a water bath using an Elmasonic P30H (37 kHz) sonicator or with a tip sonication using an Ultrasonic Processor 505 (500 Watts) and a probe of 3 mm. The Spectra/Por® dialysis membrane (standard RC tubing MWCO: 12–14 kDa) was purchased from SpectrumLabs. For filtration of the CNT suspensions, PTFE membranes (0.1 and 0.45 μm) from Millipore were used. The Boc-mono-protected TEG diamine derivative (*tert*-butyl {2-[2-(2-aminoethoxy)ethoxy]ethyl}carbamate) was synthesized using a protocol previously reported [51].

### 2.1 Peptide synthesis

Peptide syntheses were performed using optimized Fmoc chemistry protocols with a multichannel peptide synthesizer [52]. Side-chain deprotection and cleavage of the peptide from the solid support were performed by treatment with reagent K (88 % TFA v/v, 2 % triisopropylsilane v/v, 5 % dithiothreitol w/v, 5 % water v/v) for 150 min at 20 °C. The VEGF receptor binding peptides (VRbp) were purified by reversed-phase HPLC (RP-HPLC) using a preparative HPLC system (Waters) on a Nucleosil C18 (1 × 30 cm) column (Macherey Nagel). The elution was achieved with a linear gradient of aqueous 0.1 % TFA (A) and 0.08 % TFA in acetonitrile (B) at a flow rate of 6 mL min^−1^ with UV detection at 230 nm. The purity of the VEGFR-binding peptide was controlled by analytical RP-HPLC (Fig. S7) and their molecular weight was assessed by LC/MS (Fig. S8). MS (ESI, *m/z*): 1967.7 [M+H]^+^ for CGGGGGGHRHTKQRHTALH and 1967.0 [M+H]^+^ for HRHTKQRHTALHGGGGGGC.

### 2.2 Synthesis of the peptide-CNTs 5 and 6

A solution of VRbp-N (CGGGGGGHRHTKQRHTALH) or VRbp-C (HRHTKQRHTALHGGGGGGC) (6.32 mg) in water (3.5 mL) was added to a suspension of the MWCNT-maleimide **4** (7 mg) in water (3.5 mL), which was previously sonicated in a water bath for 1 min (Fig. S1). After 24 h, the nanotubes were filtered (0.1 μm Millipore membrane), dispersed in water (50 mL), sonicated for 1 min in a water bath, and filtered again. This washing process was repeated 4 times with water. The CNTs were dispersed in water and dialyzed against water for 48h to yield the peptide-CNT conjugates **5** (CNT-VRbp) and **6** (VRbp-CNT) (5 mg) after drying under vacuum. All designs are depicted in Fig. 1d, S1, and S2.

### 2.3 Synthesis and characterization of the functionalized MWCNTs 11 and MWCNTs 12

The methods employed for the production and characterization of the MWCNTs utilized in this study are comprehensively detailed in the Supplementary Information. Briefly, a solution of VRbp-N or VRbp-C **(**366 μg) in water (1.5 mL) was added to a suspension of the functionalized MWCNTs **10** (3 mg) in water (1.5 mL), which was previously sonicated in a water bath for 1 min (Fig. S2). After 24 h, the nanotubes were filtered (0.1 μm Millipore membrane), dispersed in water (50 mL), sonicated for 1 min in a water bath, and filtered again. This process was repeated 4 times with water. The MWCNTs were dispersed in water and dialyzed against water for 2 days to yield the functionalized MWCNTs **11** (Cy5-CNT-VRbp) and MWCNTs **12** (VRbp-CNT-Cy5) (2.5 mg) after drying under vacuum.

### 2.4 Cell culture

B16-F10 murine malignant melanoma cells (ATCC, CRL-6475) were cultured in Iscove's Dulbecco's modified medium (IMDM) with 10 % fetal bovine serum (FBS) and 100 μg/mL gentamycin. Human umbilical endothelial cells (HUVEC, ATCC, CRL-1730) were cultured in endothelial cell medium supplemented with 10 % fetal bovine serum, 1 % endothelial cell growth supplement (ECGS), and 100 U/mL penicillin and 100 μg/mL streptomycin. Mouse fibroblasts NIH-3T3 (ATCC, CRL-1658) and mouse macrophage J774 (ATCC, TIB-67) were cultured in DMEM supplemented with 10 % FBS and 100 U/mL gentamicin. All of them were cultured at 37 °C in 5 % CO_2_. The methods for culturing and analyzing HUVEC, NIH-3T3, and J774 are thoroughly outlined in the Supplementary Information. In the primary cultures, tissues derived from lung metastases were dissected and mechanically dissociated and the cells were cultured in IMDM medium supplemented with 10 % fetal bovine serum with antibiotics. After 7–10 days of growth, cultures were treated with 100 μg/mL of CNTs, 10 μg/mL of Taxol®, or a combination of both. Cultures were fixed 72 h after treatment using 4 % paraformaldehyde. Where indicated, microtubules were immunostained with a B512 anti-α-tubulin antibody, VEGF receptor with an anti-VEGF receptor polyclonal antibody, actin was stained with phalloidin–tetramethylrhodamine B isothiocyanate, and DNA with the Hoechst dye. Alexa Fluor 488 conjugated-secondary antibodies were goat anti-mouse IgG or anti-rabbit IgG antibodies. Fluorescent images were obtained with a Nikon A1R confocal microscope. All images were pseudo-colored.

### 2.5 Preclinical model and treatments

*In vivo* experiments were designed and performed to minimize the use of animals. Ethical approval for animal experiments was obtained before the research by the Gobierno de Cantabria, Consejería de Medio Rural, Pesa y Alimentación (accreditation number: PI-05-23). C57BL/6 mice (8 weeks old) were purchased from Janvier Labs and were housed upon arrival at the Experimentation Service (SEEA) of the University of Cantabria for 4 weeks with a 12 h light/dark cycle with free provision of food and water. Animals were maintained, handled, and sacrificed following the directive 2010/63/UE. Metastases were produced upon intravenous injection of 100.000 B16-F10 malignant melanoma cells as previously described [53,54]. For the treatment phase, 10 days after the transplantation of melanoma cells, mice were randomly divided into 6 groups, each consisting of 9 mice. These groups received the following treatments: PBS (controls), ox-MWCNTs **1**, CNT-VRbp **5**, VRbp-CNT **6**, VRbp-N, and VRbp-C. Mice were treated 3 times every 3 days at a concentration of 100 μg of CNTs (each dose) and the same amount of free peptide as that bound to the CNT-VRbp **5** or the VRbp-CNT **6**. The mice were euthanized 20 days after the transplant and their tissues were collected and fixed in formalin. The Taxol® experiment involved randomly assigning mice into groups of nine animals each. These groups received the following treatments: PBS (controls), ox-MWCNTs **1**, Taxol®, and VRbp-CNT **6** + Taxol®. This administration took place ten days following the intravenous transplant of melanoma cells. The animals were treated 3 times every 3 days at a concentration of 100 μg of CNTs and 2 mg/kg of Taxol® (each dose). The mice were euthanized 20 days after the transplant and their tissues were collected and fixed in formalin.

### 2.6 Biochemical and hematological analyses

Mice received treatment three times, with three-day intervals, at a concentration of 100 μg of CNTs. Blood samples were collected at two distinct time points: 6 days and 20 days following the initial injection of CNTs. Following anesthetization, blood specimens were immediately collected using heparinized syringes, and transferred into tubes. Blood was collected in EDTA (10 %). Serum was collected after clotting (30 min) and centrifugation at 750 *g* for 10 min. The serum then was pipetted to a new tube and stored at 4 °C until ready for analysis. These collected samples were then sent to the Dynanimed (Madrid, Spain) for both biochemical and hematological analyses.

### 2.7 Survival experiment

B16-F10 malignant melanoma cells were transplanted and 10 days later, mice were randomly assigned to six groups of five animals each. They received treatments every three days as follows: ox-MWCNTs **1**, VRbp-C, Taxol® (TX), VRbp-CNT **6**, VRbp-CNT **6** + TX. Survival of the animals was monitored from the time of cell transplantation until the occurrence of natural death. Body weight was recorded bi-weekly. Following established bioethical criteria, the animals were humanely euthanized to prevent unnecessary suffering. Survival rate comparisons were performed using Kaplan-Meier analysis alongside a log-rank test.

### 2.8 Tissue processing

Dissected tissues fixed in 10 % formalin were embedded in paraffin and sliced into 4 μm thick sections. These sections were subsequently deparaffinized and stained with hematoxylin-eosin. Fresh metastatic tissues were frozen and preserved in an optimal cutting temperature compound (OCT) for subsequent cryostat sectioning. Cryostat 6 μm-thick tissue sections were fixed in 4 % paraformaldehyde for further processing.

### 2.9 Raman spectroscopy localization of MWCNTs

The unpolarized Raman spectra were taken at room temperature with a Horiba T64000 triple spectrometer in the backscattering geometry, using the 514-nm line of a Coherent Innova Spectrum 70C Ar^+^–Kr^+^ laser and a nitrogen-cooled CCD (Jobin-Yvon Symphony) coupled to a confocal Raman microscope for detection. The laser beam was focused down to 1 μm spot at 100x objective and kept the power on the sample below 2 mW to avoid laser-heating effects on the probed material and the concomitant softening of the observed Raman peaks. The spatial resolution is diffraction-limited. In the setup used, features at r = 0.61 × 514/0.9 = 0.35 μm can be distinguished and completely resolved at a distance of 2r = 0.70 μm. Mapping was done for the stack scan with a step size of 10 μm in X/Y and a constant Z (depth) in a square of 70 × 60 μm on X/Y. To get a better signal-to-noise ratio, every spectrum was acquired with an integration time of 30 s, meaning 7 × 6 = 42 spectra for one image. The image was constructed by mapping the intensity of the G mode (1500–1700 cm^−1^). Raman quantification was done as in previous studies [[20], [34]] by the normalization of the intensities of the G band of the ox-MWCNTs of CNT-Cy5 **7**, Cy5-CNT-VRbp **11**, VRbp-CNT-Cy5 **12**, and the intensities of the D band.

### 2.10 Fluorescent MWCNT biodistribution

Animals receiving CNT-Cy5 **7**, Cy5-CNT-VRbp **11**, and VRbp-CNT-Cy5 **12** every three days intravenously were euthanized 20 days after melanoma cell transplant. The lungs and main organs were dissected, fixed, and subjected to imaging using an IVIS® imaging system. The Cy5 fluorescence signal was captured using excitation/emission wavelengths of 650/670 nm during a 1-min exposure. The fluorescence intensity was quantified as the mean radiant efficiency (photons s·cm^−2^·sr)/(μW·cm^−2^). As a baseline, mice treated with the *as-produced* ox-MWCNTs **1** were employed as a negative control for background fluorescence. Confocal microscopy imaging was conducted with identical settings for both control and experimental samples, utilizing a Nikon AIR microscope. Quantification of fluorescence was carried out using ImageJ software on confocal microscopy images obtained with identical settings. All confocal images are pseudo-colored.

### 2.11 Quantitative analysis of pulmonary metastasis

A total of 45 whole lungs, fixed in formalin, were subjected to image analysis. This equates to 90 samples when accounting for both sides. Lung images were captured using the ZEISS Stemi 508 stereo microscope for quantification, with ImageJ software utilized for this purpose. The results are presented as percentages of the overall lung area as explained in the Supplementary Information. The precise experimental techniques for quantifying the vasculature within metastatic tumors were carried out on sections stained with hematoxylin-eosin. Imaging of these tumor sections was conducted using confocal microscopy, as depicted in the supplementary figures and elaborated upon in the supplementary experimental methods.

### 2.12 Statistical analysis of pulmonary metastasis

Results are expressed as mean values along with their corresponding standard deviations (SD). Statistical differences in means were assessed using a *t-*test or ANOVA, conducted through SPSS software, and significance was established at *p* < 0.05. In cases where ANOVA yielded significant results, pairwise comparisons were carried out. The total number of events and the confidence levels achieved in the experiment (*n*) are all specified in each figure caption for reference.

## 3 Results

### 3.1 Functionalization, characterization, and biocompatibility of the CNTs

To enhance the targeting ability of CNTs and minimize premature elimination upon intravenous injection, we employed a functionalization strategy by attaching the HRHTKQRHTALH peptide to the surface of the nanotubes. This VEGFR binding peptide (VRbp) has been previously reported to bind with high affinity to the receptor of VEGF, efficiently inhibiting neovascularization in a rat corneal model [50]. Based on molecular docking experiments (Fig. S3), and considering the steric hindrance that the CNTs may exert in the binding of the peptide to the receptor, we decided to attach this peptide to the nanotubes by its two ends through a flexible glycine-rich linker terminated with a cysteine, corresponding to CGGGGGG and GGGGGGC, respectively [55]. The peptide with the C-terminal linker was designated as VRbp-C, while the one with the N-terminal linker was termed VRbp-N. Correspondingly, the CNT conjugates were named VRbp-CNT and CNT-VRbp respectively (Fig. 1).

The first step of the synthesis consisted of the chemical modification of MWCNTs with a triethylene glycol (TEG) chain and a maleimide group to allow the conjugation of the peptides (Fig. S1). The final peptides containing 19 amino acids were synthesized by solid-phase synthesis. We exploited the thiol-ene reaction between the maleimide moiety and the free thiol group of the cysteine present either at the C- or N-terminus of the two peptides. Maleimides are commonly used in the preparation of bioconjugates because proteins and peptides have cysteine residues that can readily react *via* their free SH with the maleimide double bond under mild conditions [56].

**Fig. 2:**
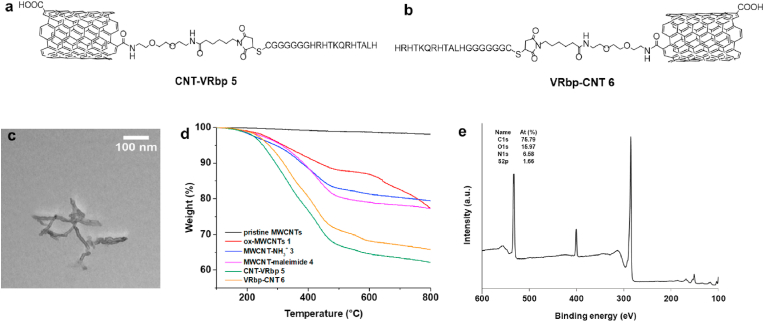
**MWCNT-peptide** synthesis and characterization**.** Chemical structure of (a) CNT-VRbp **5** and (b) VRbp-CNT **6**. (c) TEM image of VRbp-CNT **6**, (d) TGA of the peptide-nanotube conjugates and their precursors performed in N_2_ atmosphere. (e) XPS survey of VRbp-CNT **6**.

Pristine MWCNTs with an average diameter of 9.5 nm were first oxidized by an acidic treatment in a mixture of H_2_SO_4_/HNO_3_ under sonication to introduce a high number of carboxylic acids, especially at the tips and the defect sites of the nanotubes [57]. This treatment improves nanotube water dispersibility. The resulting oxidized MWCNTs (ox-MWCNTs, **1**) were further shortened by a tip sonication treatment. Their length was measured by transmission electron microscopy (TEM) and corresponded to 122 nm on average, with a range between 50 and 200 nm (Fig. S4). The carboxylic groups were activated as acyl chlorides and derivatized with a *tert*-butyloxycarbonyl (Boc)-mono-protected TEG diamine. The Boc group was subsequently removed under acidic conditions, affording MWCNT-NH_3_^+^ **3**. Next, the amine functions were coupled to a maleimide linker terminated with a carboxylic acid, in the presence of coupling reagents, to yield MWCNT-maleimide **4**. The VEGF peptides were reacted with the MWCNTs to produce the final peptide-MWCNT conjugates, namely CNT-VRbp and VRbp-CNT (Fig. 2a and b).

Functionalized MWCNTs were characterized by different techniques, including TEM, thermogravimetric analysis (TGA), X-ray photoelectron spectroscopy (XPS), and dynamic light scattering. The TEM images evidenced that both CNT-peptide conjugates were well-dispersed and mostly individualized, while their morphology did not change after the functionalization (Fig. 2c and S5a). Photographs of the suspensions of the CNT-peptide conjugates in PBS and hydrodynamic size distribution show their dispersibility (Fig. S5). For further details, please refer to the supplementary methods and Figs. S6–S12.

TGA performed under a nitrogen atmosphere was used to estimate the degree of functionalization of the MWCNTs (Fig. 2d). The pristine MWCNTs were stable over a wide temperature range (100–800 °C), while ox-MWCNTs showed a weight loss of 16 % due to the elimination of the oxygenated groups. We observed a gradual increase in weight loss after introducing the TEG linker and the maleimide, confirming the occurrence of the covalent functionalization reactions. The number of amines was estimated by the Kaiser test, affording a loading of 250 μmol per gram of MWCNT-NH_3_^+^ **3**, which drastically diminished after the functionalization with the maleimide (20 μmol/g). The conjugation of the VEGF peptides was confirmed by a higher weight loss (14.7 and 11.2 %, respectively) in comparison to the MWCNT-maleimide **4**. These values correspond to a loading of 76 and 58 μmol/g of CNT-VRbp **5** and VRbp-CNT **6**, respectively. The different conjugates were also analyzed by XPS. The XPS survey of ox-MWCNTs **1** showed that the tubes contain 88.8 and 11.2 % of carbon and oxygen, respectively (Fig. S6a). The introduction of the amino-TEG chain was confirmed by the appearance of the N1s peak (2.21 %) at ∼400 eV, corresponding to primary amines (Fig. S6b). The additional peak of S2p at 164 eV appearing in the XPS of the CNT-VRbp **5** and VRbp-CNT **6** (1.6 and 1.7 %, respectively) was indicative of a successful peptide conjugation (Fig. 2e, S6c).

Following this, the biocompatibility of the prepared MWCNTs **1** was meticulously assessed in healthy mice over a period of 8 months. Thorough evaluations, encompassing body weight measurements and histopathological examination of tissues, demonstrated no signs of toxicity within the duration of the experiment or in extended periods (see Fig. S13).

### 3.2 CNT targeting metastatic tissue

**Fig. 3:**
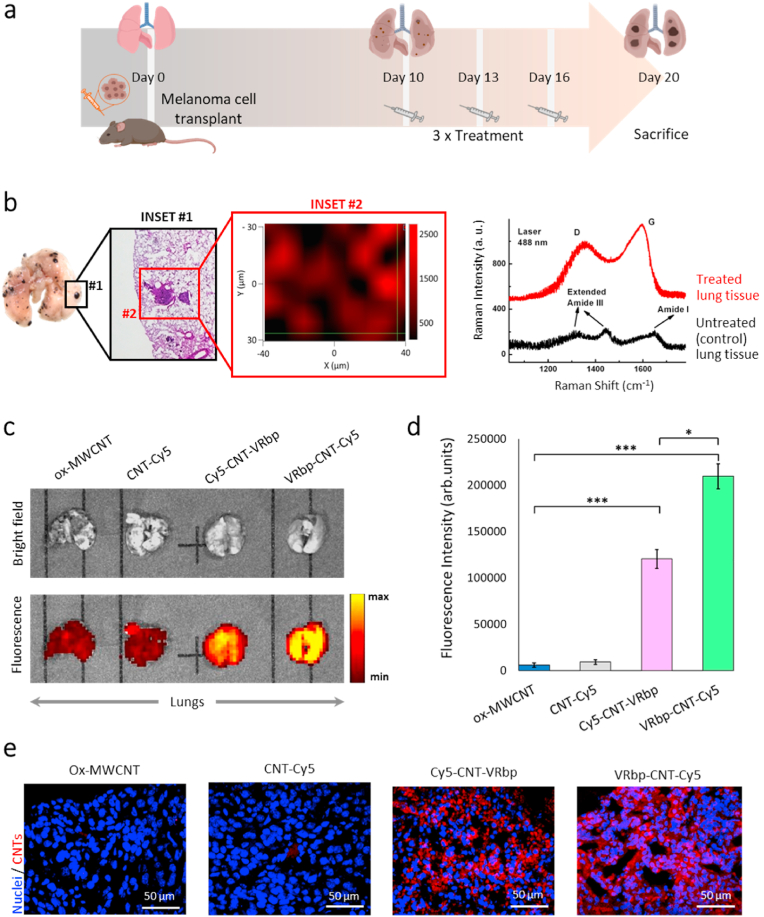
**Preclinical model and treatment approach: validation of the targeting strategy.** (a) Illustration of the experimental design for the metastasis preclinical model and treatment strategy. (b) Identification of the targeted CNTs in lung metastatic tissue through Raman spectroscopy. Inset #2 displays a single Z-plane confocal Raman image of the lung tissue, highlighting the intensity mapping of the G mode (1500-1700 cm^−1^) specific to ox-MWCNTs. (far right) Raman spectra of untreated (black) and treated (red) lung tissue exhibit characteristic ox-MWCNT fingerprints corresponding to the D and G bands. (c) IVIS® fluorescence images of the whole lungs of mice injected with ox-MWCNTs, CNT-Cy5, Cy5-CNT-VRbp, and VRbp-CNT-Cy5. (d) Quantification of fluorescence intensity from various confocal microscopy images of metastatic lung sections in mice injected with ox-MWCNTs, CNT-Cy5, Cy5-CNT-VRbp, and VRbp-CNT-Cy5. Mean ± SD, *n* = 6, ***p < 0.001 and *p < 0.01. (e) Representative confocal microscopy images depicting the Cy5 fluorescence (red channel) of the targeted nanotubes within the lung tissue. Nuclei are labeled with Hoechst (blue).

To investigate the *in vivo* effects of the targeted nanotubes in the *in vivo* context, we employed a widely recognized preclinical murine model of metastatic melanoma [58]. In the experiment, mice were intravenously injected with 1 × 10^5^ B16-F10 melanoma cells. Following an incubation period of approximately 10 days, these cells metastasized to the lung tissues, resulting in the formation of small melanoma spots visible as black-pigmented areas (Fig. 3a). To validate the existence of neovasculature within the metastatic region, immunostaining was conducted on lung tissues utilizing an anti-VEGFR antibody. Confocal microscopy images (Fig. S14) unveiled an abundance of cells expressing the VEGFR in the proximity of the developing metastasis.

Melanoma-transplanted mice were treated with the two peptide-CNT conjugates (CNT-VRbp and VRbp-CNT) following the protocol outlined in Fig. 3a. The administration of the nanotubes was performed intravenously on days 10, 13, and 16 after the transplantation of the melanoma cells. On day 20, the mice were euthanized, and their tissues were collected for anatomopathological evaluation. Three distinct techniques were utilized to confirm the targeting efficiency of both CNT-VRbp and VRbp-CNT conjugates toward metastatic vasculature. These included Raman spectroscopy (for detecting the specific signature of ox-MWCNTs), *in toto* fluorescent detection to identify the fluorescence in the entire organ, and confocal microscopy.

The confirmation of targeting the tumor microenvironment was extended by employing a fluorescent dye, cyanine 5 (Cy5), to label the nanotubes (Fig. S2). We compared the distribution of two nanotube-peptide conjugates, namely Cy5-CNT-VRbp **11**, and VRbp-CNT-Cy5 **12**, with nanotube controls that lacked the targeting peptide, referred to as CNT-Cy5 **7.** The distribution of the fluorescent nanotubes was investigated at both, macroscopic and microscopic levels. Macroscopically, the emission of the Cy5 dye was detected using an IVIS® imaging system. The estimation of the fluorescence signal in intact lungs indicated that, in comparison to the distribution of the untargeted control nanotubes (ox-MWCNTs **1**, and the CNT-Cy5 **7**), nearly 50 % of the VRbp-bound nanotubes in the body accumulated in the metastatic lungs (Fig. 3c). Interestingly, the targeting efficiency of the VRbp-CNT-Cy5 **12** conjugate shows a slight improvement in the intensity of fluorescence compared to Cy5-CNT-VRbp **11** (Fig. S16).

When the sections of the lungs from treated mice were subjected to Raman spectroscopy, the acquired spectra obtained from mice injected with MWCNTs revealed the distinctive Raman tangential G band and Amide I band, as illustrated in Fig. S15 [59]. The semi-quantitative evaluation comparing VRbp-targeted nanotubes with untargeted CNT-Cy5 in metastatic lungs revealed that the targeted MWCNTs were approximately four times more abundant in the metastatic lung tissue. This outcome signifies a notably greater accumulation of VRbp-targeted nanotubes in the metastatic lung tissue compared to the untargeted nanotubes.

In addition, Raman confocal imaging was employed to determine the localization of nanotubes within the metastatic tumor microenvironment. Fig. 3b (inset 2) displays a representative single-Z plane image of a 60 × 80 μm region from a lung tissue section containing melanoma metastasis. This image illustrates the identification and quantification of distinctive MWCNT fingerprints (indicated by the red line), including the D and G bands, in comparison to untreated lung tissue (represented by the black line).

Fig. S2 Fig. S16

Metastatic lung tissue samples also underwent confocal microscopy imaging, with identical settings, to enable automated quantitative analysis of the fluorescent signal in the sections. This quantification of the fluorescent signal within the lung tissues confirmed a substantial increase in the Cy5 signal when the nanotubes were targeted with the VRbp ligand peptide. In line with the previous observations, linking the nanotube via the C-terminus of the peptide (VRbp-CNT-Cy5 **12**) further enhanced the targeting effectiveness (Fig. 3d). Representative confocal fluorescence microscopy images can be observed in Fig. 3e.

### 3.3 Improved survival associated with the anti-metastatic effect of the CNT conjugates

After establishing the affinity of the peptide-CNT conjugates for metastatic lung tissues, our subsequent goal was to validate the treatment. To achieve this, we administered various treatments to a total of 45 mice bearing metastases (Materials and Methods). As negative controls, we administered: (i) PBS, (ii) the *as-prepared* ox-MWCNTs **1**, and (iii) the two peptides, modified either at the C- or N-terminus (referred to as VRbp-N and VRbp-C). The treatment plan included administering three intravenous doses of the nanotube conjugates and controls. Ten days after initiating the treatment, the mice with metastases were euthanized (as depicted in Fig. 3a).

**Fig. 4:**
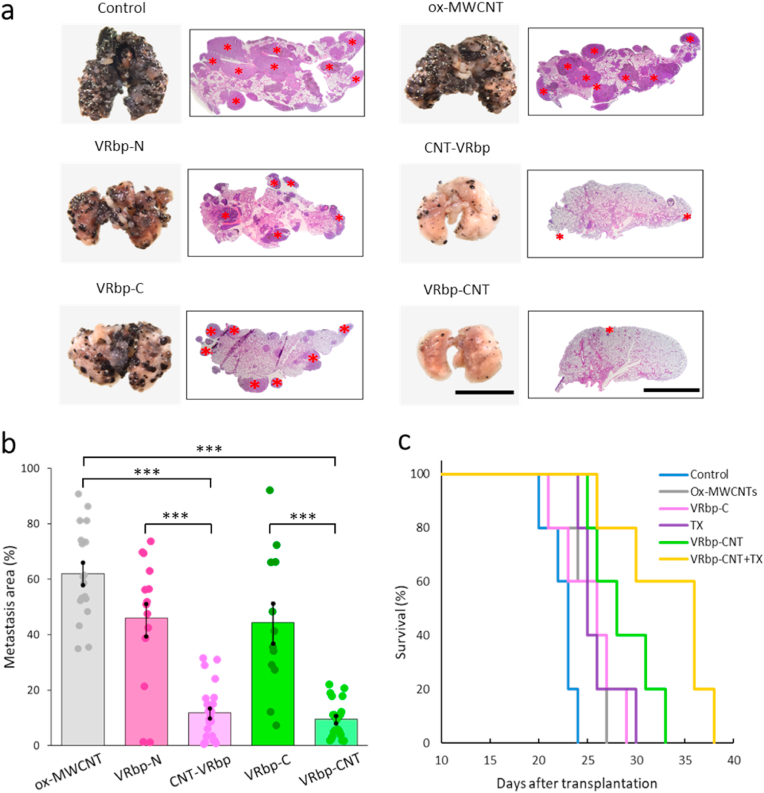
**Anti-metastatic effect of the targeted CNTs.** (a) Images of whole metastasis-bearing lungs and corresponding histological sections. Asterisks have been marked on the metastatic sites within the lung parenchyma. The scale bars represent 10 and 2 mm, respectively. (b) Quantification of the percentage of the lung parenchyma affected by metastasis (as a % of the total surface compared to control mice). Statistical analysis showed significant differences (*n* = 90, *p* = 0, 0, 6.71 E−07 and 2.74 E−07 comparing ox-MWCNTs to CNT-VRbp, ox-MWCNTs to VRbp-CNT, VRbp-N to CNT-VRbp and VRbp-C to VRbp-CNT, respectively; ***p < 0.001). (c) Survival curve for the metastasis-bearing mice. Kaplan-Meier survival graph showing improved survival in mice. The differences in survival between the groups treated with ox-MWCNTs **1**, VRbp-C, and TX are also statistically significant from those of the group treated with VRbp-CNT **6** + TX (*p <* 0.01).

Throughout the treatment, we conducted assessments including body weight measurements, histological examination of tissues, as well as blood biochemical and hematological analyses. Notably, no discernible differences were observed in these parameters (Fig. S17, and Tables S1 and S2). Following the treatment and subsequent sacrifice of the animals, a macroscopic anatomopathological examination was carried out. The peptide-nanotube conjugates demonstrated an outstanding anti-metastatic effect, surpassing the efficacy observed in all control groups. Particularly noteworthy were the lungs of animals treated with both CNT-VRbp **5** and VRbp-CNT **6**, which displayed only a minimal presence of small melanoma colonies, as indicated by the red asterisks in Fig. 4a.

Automatic image analysis was employed to quantify the extent of lung area affected by metastasis in a total of 45 lungs (2 sides) (Fig. S18). The quantification was conducted by comparing the treated groups with the untreated controls (injected with PBS), as well as mice injected with the *as-prepared* ox-MWCNTs, or the two peptides (VRbp-N, VRbp-C). The results, as seen with the naked eye, confirmed that both the *as-prepared* ox-MWCNTs and the two VEGF receptor-binding peptides reduced metastasis by approximately 40–55 % when compared to PBS-treated controls (Fig. 4b). Both peptide-nanotube conjugates were able to reduce the surface area of the lung affected by metastasis by approximately 90 %. The full statistical analysis (+/-SD mean/*DF*/*p*) is shown in Table S3. Interestingly, both peptide configurations produced comparable results in terms of the metastatic area affected. However, the VRbp-CNT **6** conjugate showed a greater reduction in metastatic area. This suggests that it may have interacted more effectively with the VEGF receptor.

Microscopic examination of tumor sections stained with hematoxylin-eosin revealed a noticeable decrease in observable blood vessels within the tumoral stroma in animals treated with the targeted nanotubes, compared to the control group (Fig. S19). The increased presence of apoptotic bodies in the tumor sections of animals treated with targeted CNTs was also highly conspicuous (green arrows). Quantification of the intratumoral vasculature in histological sections of the metastatic areas confirmed a statistically significant reduction in angiogenesis in lungs from animals treated with the targeted nanotubes (Fig. S20). Parallel standard *in vitro* assays utilizing HUVEC cells as a model system for studying endothelial cell processes also corroborated targeted carbon nanotube accumulation in the endothelial cells and a negative effect on angiogenesis (Fig. S21)

Finally, we assessed the treatment's impact on the animals' lifespan. The results unequivocally revealed substantial survival benefits resulting from the targeted nanotube treatment, surpassing the survival rates observed in mice treated with control interventions or left untreated (p < 0.01) (Fig. 4c).

### 3.4 Combination effect of the targeted CNT conjugates with a traditional chemotherapy

**Fig. 5:**
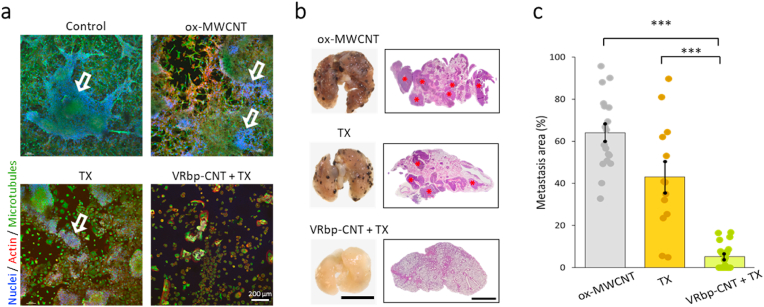
**Synergistic effects of VRbp-CNT in combination with Taxol®.** (a) Images of mixed primary cultures of lung metastatic tissues treated with ox-MWCNTs (100 μg/mL), Taxol® (TX, 2 μg/mL), and the combination of VRbp-CNT and TX (100 μg/mL and TX, 2 μg/mL each). Cultures treated with nanotubes and chemotherapy exhibit a noticeable reduction in both the number and size of melanoma cell colonies, as indicated by white arrows. (b) Representative images and histological sections of lungs bearing metastases from animals treated with ox-MWCNTs (3 × 100 μg/animal), TX (3 × 2 mg/kg), or both (VRbp-CNT + TX). Significantly fewer melanoma metastases are observed in the lungs (red asterisks) treated with the combination of VRbp-CNT and Taxol®. The scale bars represent 10 and 2 mm, respectively. (c) Quantification shows the percentage of lung parenchyma affected by metastasis, presented as a percentage of the total surface compared to PBS-treated control mice (as a % of the total surface compared to PBS-treated control mice) (*n =* 52, *p* = 0 and *p* = 2.04 E−07 comparing ox-MWCNTs to VRbp-CNT and TX to VRbp-CNT + TX, respectively; ***p < 0.001).

Some studies have demonstrated that combining MWCNTs with microtubule-binding chemotherapy agents has an adjuvant effect, as both treatments disrupt cellular cytoskeleton rearrangements during proliferation [34]. Building on this knowledge, we set out to investigate whether the anti-metastatic effect of MWCNTs could be further enhanced when used in combination with Taxol® administered separately. This drug, also known as paclitaxel, is a well-established microtubule dynamics inhibitor drug used in chemotherapy. As a preliminary test, we employed mixed primary cultures of metastatic lung cells obtained from dissected melanoma metastasis. The cultures were subjected to separate treatments with the *as-prepared* ox-MWCNTs **1** and Taxol®, and the observed effects were then compared to cultures exposed to the targeted nanotubes (VRbp-CNT **6**) and the drug alone. Consistent with expectations, a noticeable additive inhibitory effect on the growth of primary mixed cultures of malignant melanoma cells was observed after 72 h of *in vitro* treatment (Fig. 5a).

In the preclinical model, administration of intravenous treatments showed that separate injection of Taxol® in addition to VRbp-CNT treatment resulted in a remarkable inhibition of metastasis. This effect was evident at both macroscopic and microscopic levels when compared to the control groups (Fig. 5b). Quantitative analysis of the affected lung areas revealed that over 95 % of the lung parenchyma was completely free from melanoma spots, and in some lungs, there was a complete absence of metastatic lesions (Fig. 5c, Table S4). These findings underscore a substantial additive effect between MWCNTs and chemotherapy (Table S5). Besides, as expected, these results were accompanied by a significant improvement in survival (*p* < 0.001, Fig. 4c).

The remarkable effectiveness of the targeted nanotubes in the metastatic vasculature, and likely also in the tumor microenvironment, suggests that the cytotoxic effect of these nanomaterials, which impedes tumor vascular neogenesis, occurs either simultaneously or following the local infiltration of Taxol® into the metastatic sites. These results provide substantial support for the potential of CNTs as adjuvant treatments to augment the efficacy of conventional anti-cancer drugs, particularly in cases of recurrent metastases or when escalating chemotherapy doses are constrained by adverse effects.

## 4 Discussion

Treating metastatic melanoma remains a formidable challenge, with numerous endeavors to enhance patient survival proving unsuccessful [60]. Cytotoxic chemotherapy has been used for over three decades but with modest and transitory anti-tumor efficacy. Newer targeted therapy and immunotherapy have improved survival but often result in short-lived responses that, as with chemotherapy, lead to resistance and relapse. So, there is a great need for new therapeutic alternatives.

CNTs have been studied as effective therapeutic tools for the treatment of metastatic cancer in both advanced diagnosis and therapeutic strategies [61] CNTs. Some have been used intravenously as nanocarriers for the delivery of drugs [[62], [63], [64]] or nucleic acids [65,66], often combining tumor-targeting ligands and anti-cancer drugs [67], or in thermal therapy [68]. More particularly, MWCNTs modified with gangliosides can block melanoma-associated metastatic events, preventing migration and invasiveness of human melanoma cells *in vitro* [69]. Also, ox-MWCNTs injected subcutaneously in tumor-bearing mice, can activate phagocytosis of resident macrophages and recruit phagocytes from other tissues, inhibiting tumor progression and metastasis in the lung [70]. Very recently, CNTs functionalized with synthetic oligodeoxynucleotides and injected intratumorally were found to suppress local xenograft tumor growth and liver metastasis, leading to 75 % of cured mice, significantly prolonging animal survival and limiting the formation of liver metastatic nodules [71]. In summary, all these experiments yield results that indicate that CNTs have enormous potential as an adjuvant anti-tumor treatment where therapies such as chemotherapy or radiology have proven to have low effectivity, and where targeted therapies have a transient effect.

Our study conducted in a preclinical model of metastatic melanoma presents a novel and effective strategy for inhibiting metastatic growth by utilizing ox-MWCNTs targeted to the tumor microenvironment. We chose this type of nanotubes because they are biodegradable [35,[40], [41], [42], [43], [44], [45], [46]]. Upon intravenous administration, these engineered nanotubes demonstrate a potent anti-metastatic effect, which is further enhanced when used in combination with traditional chemotherapies as adjuvants. The results of this study support the hypothesis that the targeting peptide directs the nanotubes to the neovasculature of the metastatic tumor, where they exert their cytotoxic effect on the endothelial cells (Fig. 1, S21). In addition to a reduction in blood supply, the nanotubes also induce a reduction in melanoma cells and the tumor microenvironment supporting cells, including endothelial cells, macrophages, and fibroblasts, all of which contribute significantly to a potent anti-metastatic effect. Although validation of this hypothesis *in vivo* is a major challenge, *in vitro* studies on standard cell lines show greater sensitivity to nanotube treatment for macrophages and fibroblasts than for malignant melanoma cells (Fig. S22). In summary, the inherent antiproliferative and cytotoxic activity of the nanotubes, coupled with their extended presence in the target tissue before local degradation by tumor-associated macrophages [35,46,72] likely plays a role in the enduring and consistent cytotoxic effect within the tumor environment.

## 5 Conclusion

In this study, we illustrate the remarkable anti-metastatic efficacy of targeted oxidized MWCNTs, known for their intrinsic anti-tumoral properties. Their synergistic application with Taxol® as adjuvants not only enhances overall effectiveness but also allows for a reduction in the drug dosage. Extended animal experiments assessing the toxicity of oxidized MWCNTs reveal minimal adverse effects, suggesting their potential for safe and effective use across diverse applications.

The effectiveness of this approach is subject to variation based on the specific characteristics of the cancer type and the tumor microenvironment. For instance, tumors characterized by robust angiogenesis, extensive neovascularization, and hematogenous spread are likely to derive the greatest benefit. Moreover, CNTs offer a potential solution to hinder resistance encountered by conventional treatments, offering a promising avenue for improving outcomes in cancer patients.

## CRediT authorship contribution statement

**Lorena García-Hevia:** Writing – original draft, Methodology, Investigation. **Rym Soltani:** Methodology, Investigation. **Jesús González:** Methodology, Investigation. **Olivier Chaloin:** Methodology, Investigation. **Cécilia Ménard-Moyon:** Writing – review & editing, Supervision, Investigation, Funding acquisition. **Alberto Bianco:** Writing – review & editing, Supervision, Methodology, Funding acquisition. **Mónica L. Fanarraga:** Writing – review & editing, Writing – original draft, Supervision, Methodology, Funding acquisition.

## Declaration of competing interest

The authors declare that they have no known competing financial interests or personal relationships that could have appeared to influence the work reported in this paper.

## References

[1] Xu M., Hu K., Liu Y., Huang Y., Liu S., Chen Y., Wang D., Zhou S., Zhang Q., Mei N., Lu H., Li F., Gao X., Chen J. (2021). Systemic metastasis-targeted nanotherapeutic reinforces tumor surgical resection and chemotherapy. Nat. Commun..

[2] Mehnert J.M., McCarthy M.M., Jilaveanu L., Flaherty K.T., Aziz S., Camp R.L., Rimm D.L., Kluger H.M. (2010). Quantitative expression of VEGF, VEGF-R1, VEGF-R2, and VEGF-R3 in melanoma tissue microarrays. Hum. Pathol..

[3] Tas F., Duranyildiz D., Oguz H., Camlica H., Yasasever V., Topuz E. (2006). Circulating serum levels of angiogenic factors and vascular endothelial growth factor receptors 1 and 2 in melanoma patients. Melanoma Res..

[4] Mahabeleshwar G.H., Byzova T.V. (2007). Angiogenesis in melanoma. Semin. Oncol..

[5] Folkman J., Klagsbrun M. (1987). Angiogenic factors. Science.

[6] Kieran M.W., Kalluri R., Cho Y.J. (2012). The VEGF pathway in cancer and disease: responses, resistance, and the path forward. Cold Spring Harb Perspect Med.

[7] Duda D.G., Batchelor T.T., Willett C.G., Jain R.K. (2007). VEGF-targeted cancer therapy strategies: current progress, hurdles and future prospects. Trends in molecular.

[8] Liu Y., Li Y., Wang Y., Lin C., Zhang D., Chen J., Ouyang L., Wu F., Zhang J., Chen L. (2022). Recent progress on vascular endothelial growth factor receptor inhibitors with dual targeting capabilities for tumor therapy. J. Hematol. Oncol..

[9] Liu G., Chen T., Ding Z., Wang Y., Wei Y., Wei X. (2021). Inhibition of FGF-FGFR and VEGF-VEGFR signalling in cancer treatment. Cell Prolif.

[10] Ellis L.M., Hicklin D.J. (2008). VEGF-targeted therapy: mechanisms of anti-tumour activity. Nat. Rev. Cancer.

[11] Binnewies M., Roberts E.W., Kersten K., Chan V., Fearon D.F., Merad M., Coussens L.M., Gabrilovich D.I., Ostrand-Rosenberg S., Hedrick C.C., Vonderheide R.H., Pittet M.J., Jain R.K., Zou W., Howcroft T.K., Woodhouse E.C., Weinberg R.A., Krummel M.F. (2018). Understanding the tumor immune microenvironment (TIME) for effective therapy. Nat. Med..

[12] Itatani Y., Kawada K., Yamamoto T., Sakai Y. (2018). Resistance to anti-angiogenic therapy in cancer-alterations to anti-VEGF pathway. Int. J. Mol. Sci..

[13] Suh Y., Amelio I., Guerrero Urbano T., Tavassoli M. (2014). Clinical update on cancer: molecular oncology of head and neck cancer. Cell Death Dis..

[14] Gray-Schopfer V., Wellbrock C., Marais R. (2007). Melanoma biology and new targeted therapy. Nature.

[15] Sandru A., Voinea S., Panaitescu E., Blidaru A. (2014). Survival rates of patients with metastatic malignant melanoma. J Med Life.

[16] van der Meel R., Sulheim E., Shi Y., Kiessling F., Mulder W.J.M., Lammers T. (2019). Smart cancer nanomedicine. Nat. Nanotechnol..

[17] Wilhelm S., Tavares A.J., Dai Q., Ohta S., Audet J., Dvorak H.F., Chan W.C.W. (2016). Analysis of nanoparticle delivery to tumours. Nat. Rev. Mater..

[18] Mahmoudi M. (2018). Debugging nano–bio interfaces: systematic strategies to accelerate clinical translation of nanotechnologies. Trends Biotechnol..

[19] Wong B.S., Yoong S.L., Jagusiak A., Panczyk T., Ho H.K., Ang W.H., Pastorin G. (2013). Carbon nanotubes for delivery of small molecule drugs. Adv. Drug Deliv. Rev..

[20] González-Lavado E., Valdivia L., García-Castaño A., González F., Pesquera C., Valiente R., Fanarraga M.L. (2019). Multi-walled carbon nanotubes complement the anti-tumoral effect of 5-Fluorouracil. Oncotarget.

[21] Battigelli A., Ménard-Moyon C., Da Ros T., Prato M., Bianco A. (2013). Endowing carbon nanotubes with biological and biomedical properties by chemical modifications. Adv. Drug Deliv. Rev..

[22] Dinesh B., Bianco A., Ménard-Moyon C. (2016). Designing multimodal carbon nanotubes by covalent multi-functionalization. Nanoscale.

[23] Rodríguez-Fernández L., Valiente R., González J., Villegas J.C., Fanarraga M.L. (2012). Multiwalled carbon nanotubes display microtubule biomimetic properties in vivo, enhancing microtubule assembly and stabilization. ACS Nano.

[24] García-Hevia L., Fernández F., Grávalos C., García A., Villegas J.C., Fanarraga M.L. (2014). Nanotube interactions with microtubules: implications for cancer medicine. Nanomedicine.

[25] García-Hevia L., Fanarraga M.L. (2020). Microtubule cytoskeleton - disrupting activity of MWCNTs: applications in cancer treatment. J. Nanobiotechnol..

[26] Garcia-Hevia L., Valiente R., Fernandez-Luna J.L., Flahaut E., Rodriguez-Fernandez L., Villegas J.C., Gonzalez J., Fanarraga M.L. (2015). Inhibition of cancer cell migration by multiwalled carbon nanotubes. Adv. Healthcare Mater..

[27] Villegas J.C., Álvarez-Montes L., Rodríguez-Fernández L., González J., Valiente R., Fanarraga M.L. (2014). Multiwalled carbon nanotubes hinder microglia function interfering with cell migration and phagocytosis. Adv. Healthcare Mater..

[28] García-Hevia L., Valiente R., González J., Terán H., Fernández-Luna J.L., Villegas J.C., Fanarraga M.L. (2015). Anti-cancer cytotoxic effects of multiwalled carbon nanotubes. Curr. Pharmaceut. Des..

[29] Ali-Boucetta H., Al-Jamal K.T., Kostarelos K. (2011). Cytotoxic assessment of carbon nanotube interaction with cell cultures. Methods Mol. Biol..

[30] Vittorio O., Raffa V., Cuschieri A. (2009). Influence of purity and surface oxidation on cytotoxicity of multiwalled carbon nanotubes with human neuroblastoma cells. Nanomedicine.

[31] Popov V.N. (2004). Carbon nanotubes: properties and application. Mater. Sci. Eng. R Rep..

[32] Sato M. (2011). Elastic and plastic deformation of carbon nanotubes. Procedia Eng..

[33] Dinu C.Z., Bale S.S., Zhu G., Dordick J.S. (2009). Tubulin encapsulation of carbon nanotubes into functional hybrid assemblies. Small.

[34] García-Hevia L., Villegas J.C., Fernández F., Casafont I., González J., Valiente R., Fanarraga M.L. (2016). Multiwalled carbon nanotubes inhibit tumor progression in a mouse model. Adv. Healthcare Mater..

[35] González-Lavado E., Iturrioz-Rodríguez N., Padín-González E., González J., García-Hevia L., Heuts J., Pesquera C., González F., Villegas J.C., Valiente R., Fanarraga M.L. (2018). Biodegradable multi-walled carbon nanotubes trigger anti-tumoral effects. Nanoscale.

[36] Jordan M.A., Wilson L. (1998). Microtubules and actin filaments: dynamic targets for cancer chemotherapy. Curr. Opin. Cell Biol..

[37] A. Poland C., Duffin R., Kinloch I., Maynard A., Wallace W.A.H., Seaton A., Stone V., Brown S., Macnee W., Donaldson K. (2008). Carbon nanotubes introduced into the abdominal cavity of mice show asbestos-like pathogenicity in a pilot study. Nat. Nanotechnol..

[38] Hansen S.F., Lennquist A. (2020). Carbon nanotubes added to the SIN list as a nanomaterial of very high concern. Nat. Nanotechnol..

[39] Heller D.A., Jena P.V., Pasquali M., Kostarelos K., Delogu L.G., Meidl R.E., Rotkin S.V., Scheinberg D.A., Schwartz R.E., Terrones M., Wang Y., Bianco A., Boghossian A.A., Cambré S., Cognet L., Corrie S.R., Demokritou P., Giordani S., Hertel T., Ignatova T., Islam M.F., Iverson N.M., Jagota A., Janas D., Kono J., Kruss S., Landry M.P., Li Y., Martel R., Maruyama S., Naumov A.V., Prato M., Quinn S.J., Roxbury D., Strano M.S., Tour J.M., Weisman R.B., Wenseleers W., Yudasaka M. (2020). Banning carbon nanotubes would be scientifically unjustified and damaging to innovation. Nat. Nanotechnol..

[40] Sureshbabu A.R., Kurapati R., Russier J., Ménard-Moyon C., Bartolini I., Meneghetti M., Kostarelos K., Bianco A. (2015). Degradation-by-design: surface modification with functional substrates that enhance the enzymatic degradation of carbon nanotubes. Biomaterials.

[41] Ali-Boucetta H., Nunes A., Sainz R., Herrero M.A., Tian B., Prato M., Bianco A., Kostarelos K. (2013). Asbestos-like pathogenicity of long carbon nanotubes alleviated by chemical functionalization. Angew. Chem. Int. Ed..

[42] Mokhtari-Farsani A., Hasany M., Lynch I., Mehrali M. (2021). Biodegradation of carbon-based nanomaterials: the importance of “biomolecular corona” consideration. Adv. Funct. Mater..

[43] Nunes A., Bussy C., Gherardini L., Meneghetti M., Herrero M.A., Bianco A., Prato M., Pizzorusso T., Al-Jamal K.T., Kostarelos K. (2012). In vivo degradation of functionalized carbon nanotubes after stereotactic administration in the brain cortex. Nanomedicine.

[44] Elgrabli D., Dachraoui W., Ménard-Moyon C., Liu X.J., Begin D., Begin-Colin S., Bianco A., Gazeau F., Alloyeau D. (2015). Carbon nanotube degradation in macrophages: live nanoscale monitoring and understanding of biological pathway. ACS Nano.

[45] Kotchey G.P., Zhao Y., Kagan V.E., Star A. (2013). Peroxidase-mediated biodegradation of carbon nanotubes in vitro and in vivo. Adv. Drug Deliv. Rev..

[46] Russier J., Oudjedi L., Piponnier M., Bussy C., Prato M., Kostarelos K., Lounis B., Bianco A., Cognet L. (2017). Direct visualization of carbon nanotube degradation in primary cells by photothermal imaging. Nanoscale.

[47] Loh K.P., Ho D., Chiu G.N.C., Leong D.T., Pastorin G., Chow E.K.H. (2018). Clinical applications of carbon nanomaterials in diagnostics and therapy. Adv. Mater..

[48] Patel K.D., Singh R.K., Kim H.W. (2019). Carbon-based nanomaterials as an emerging platform for theranostics. Mater. Horiz..

[49] Soltani R., Guo S., Bianco A., Ménard‐Moyon C. (2020). Carbon nanomaterials applied for the treatment of inflammatory diseases: preclinical evidence. Adv. Ther..

[50] Zhang Y., He B., Liu K., Ning L., Luo D., Xu K., Zhu W., Wu Z., Huang J., Xu X. (2017). A novel peptide specifically binding to VEGF receptor suppresses angiogenesis in vitro and in vivo. Signal Transduct. Targeted Ther..

[51] Pastorin G., Wu W., Wieckowski S., Briand J.P., Kostarelos K., Prato M., Bianco A. (2006). Double functionalisation of carbon nanotubes for multimodal drug delivery. Chem. Commun..

[52] Neimark J., Briand J.P. (1993). Development of a fully automated multichannel peptide synthesizer with integrated TFA cleavage capability. Pept. Res..

[53] Gautam A., Waldrep J.C., Densmore C.L., Koshkina N., Melton S., Roberts L., Gilbert B., Knight V. (2002). Growth inhibition of established B16-F10 lung metastases by sequential aerosol delivery of p53 gene and 9-nitrocamptothecin. Gene Ther..

[54] Valdivia L., García-Hevia L., Bañobre-lópez M., Gallo J., Valiente R., Fanarraga M.L. (2021). Solid-lipid particles for lung metastasis treatment. Pharmaceutics.

[55] Ning L., Li Z., Bai Z., Hou S., He B., Huang J., Zhou P. (2018). Computational design of antiangiogenic peptibody by fusing human IgG1 Fc fragment and HRH peptide: structural modeling, energetic analysis, and dynamics simulation of its binding potency to VEGF receptor. Int. J. Biol. Sci..

[56] Battigelli A., Russier J., Venturelli E., Fabbro C., Petronilli V., Bernardi P., Da Ros T., Prato M., Bianco A. (2013). Peptide-based carbon nanotubes for mitochondrial targeting. Nanoscale.

[57] Samorì C., Sainz R., Ménard-Moyon C., Toma F.M., Venturelli E., Singh P., Ballestri M., Prato M., Bianco A. (2010). Potentiometric titration as a straightforward method to assess the number of functional groups on shortened carbon nanotubes. Carbon.

[58] Overwijk W.W., Restifo N.P. (2001). B16 as a mouse model for human melanoma. Curr. Protoc. Im..

[59] Dresselhaus M.S., Dresselhaus G., Saito R., Jorio A. (2005). Raman spectroscopy of carbon nanotubes. Phys. Rep..

[60] Arasi M.B., Pedini F., Valentini S., Felli N., Felicetti F. (2020). Advances in natural or synthetic nanoparticles for metastatic melanoma therapy and diagnosis. Cancers.

[61] Sheikhpour M., Golbabaie A., Kasaeian A. (2017). Carbon nanotubes: a review of novel strategies for cancer diagnosis and treatment. Mater. Sci. Eng. C.

[62] Z. Liu, K. Chen, C. Davis, S. Sherlock, Q. Cao, X. Chen, Drug delivery with carbon nanotubes for in vivo cancer treatment, Cancer Res. 68 (2009)6652–6660. 10.1158/0008-5472.CAN-08-1468 PMC2562710 18701489

[63] Qi X., Rui Y., Fan Y., Chen H., Ma N., Wu Z. (2015). Galactosylated chitosan-grafted multiwall carbon nanotubes for pH-dependent sustained release and hepatic tumor-targeted delivery of doxorubicin in vivo. Colloids Surf. B.

[64] Liu Z., Fan A.C., Rakhra K., Sherlock S., Goodwin A., Chen X., Yang Q., Felsher D.W., Dai H. (2009). Supramolecular stacking of doxorubicin on carbon nanotubes for in vivo cancer therapy. Angew. Chem. Int. Ed..

[65] Podesta J.E., Al-Jamal K.T., Herrero M.A., Tian B., Ali-Boucetta H., Hegde V., Bianco A., Prato M., Kostarelos K. (2009). Antitumor activity and prolonged survival by carbon-nanotube-mediated therapeutic siRNA silencing in a human lung xenograft model. Small.

[66] Guo C., Al-Jamal W.T., Toma F.M., Bianco A., Prato M., Al-Jamal K.T., Kostarelos K. (2015). Design of cationic multiwalled carbon nanotubes as efficient siRNA vectors for lung cancer xenograft eradication. Bioconjugate Chem..

[67] Bhirde A.A., Patel V., Gavard J., Zhang G., Sousa A.A., Masedunskas A., Leapman R.D., Weigert R., Gutkind J.S., Rusling J.F. (2009). Targeted killing of cancer cells in vivo and in vitro with EGF-directed carbon nanotube-based drug delivery. ACS Nano.

[68] Moon H.K., Lee S.H., Choi H.C. (2009). In vivo near-infrared mediated tumor destruction by photothermal effect of carbon nanotubes. ACS Nano.

[69] Arosio P., Comito G., Orsini F., Lascialfari A., Chiarugi P., Ménard-Moyon C., Nativi C., Richichi B. (2018). Conjugation of a GM3 lactone mimetic on carbon nanotubes enhances the related inhibition of melanoma-associated metastatic events. Org. Biomol. Chem..

[70] Yang M., Meng J., Cheng X., Lei J., Guo H., Zhang W., Kong H., Xu H. (2012). Multiwalled carbon nanotubes interact with macrophages and influence tumor progression and metastasis. Theranostics.

[71] Jin H., Gao S., Song D., Liu Y., Chen X. (2021). Intratumorally CpG immunotherapy with carbon nanotubes inhibits local tumor growth and liver metastasis by suppressing the epithelial-mesenchymal transition of colon cancer cells. Anti Cancer Drugs.

[72] Bussy C., Hadad C., Prato M., Bianco A., Kostarelos K. (2016). Intracellular degradation of chemically functionalized carbon nanotubes using a long-term primary microglial culture model. Nanoscale.

