# Supplementary material for: Carbon nanotubes targeted to the tumor microenvironment inhibit metastasis in a preclinical model of melanoma
Source: Bioact Mater. 2023 Dec 28;34:237–47. doi: 10.1016/j.bioactmat.2023.12.013 (PMC10787223; doi:10.1016/j.bioactmat.2023.12.013)
Supplement: Multimedia component 1 [file mmc1.docx]

**SUPPLEMENTARY INFORMATION**

**BIOMAT 1587**

# Carbon nanotubes targeted to the tumor microenvironment inhibit metastasis in a preclinical model of melanoma

List of the Supplementary information:

Supplementary Methods:

- Analytical techniques.
- Synthesis of Boc-amino PEG-Cy5 linker.
- Synthesis of NH_3_^+^-PEG-Cy5 linker.
- Synthesis of MWCNTs 1.
- Synthesis of MWCNTs 2.
- Synthesis of MWCNT-NH_3_^+^ 3.
- Synthesis of MWCNT-maleimide 4.
- Synthesis of functionalized MWCNTs 7-10.
- Evaluation of ox-MWCNTs in angiogenesis.
- Effect of ox-MWCNTs on cells.

Supplementary Figures:

**Figure S1.** Synthesis of the peptide-CNT conjugates.

**Figure S2.** Synthesis of the fluorescent peptide-CNT conjugates **11** and **12**.

**Figure S3**. The 3D structure of the VEGF receptor-binding peptide (VRbp).

**Figure S4.** Length distribution of ox-MWCNTs **1**.

**Figure S5.** CNT characterization by TEM and DLS.

**Figure S6.** XPS of ox-MWCNTs **1**, MWCNT-NH_3_^+^ **3**, and CNT-VRbp **5**.

**Figure S7. HPLC chromatograms of VRbp-N and VRbp-C.**

**Figure S8. Mass spectrometry data of the peptides.**

**Figure S9. HPLC of NH_3_^+^-PEG-Cy5 linker.**

**Figure S10. Mass spectrometry of NH_3_^+^-PEG-Cy5 linker.**

**Figure S11.** TGA of the peptide-CNT-Cy5 conjugates.

**Figure S12.** Fluorescence spectra of the peptide-CNT conjugates **11** and **12**.

**Figure S13.** Toxicity assay.

**Figure S14.** Images of metastatic lung tissue captured using both fluorescent confocal microscopy and phase contrast microscopy.

**Figure S15.** Raman spectra of lung tissue treated with CNTs.

**Figure S16.** CNT targeting.

**Figure S17.** Animal monitoring studies.

**Figure S18.** Lung metastasis quantification.

**Figure S19.** Micrographs of tumor paraffin sections stained with hematoxylin and eosin.

**Figure S20.** Intratumoral vascular study.

**Figure S21**. *In vitro* angiogenesis inhibition test.

**Figure S22.** Phase-contrast images of cultures of murine malignant melanoma cells, NIH-3T3 murine fibroblasts, and BV2 murine macrophages treated with CNTs.

Supplementary Tables:

**Table S1.** Biochemical blood parameters in treated animals and controls.

**Table S2.** Hematological parameters in treated animals and controls.

**Table S3.** Values of metastasis-affected lung parenchyma area following different intravenous treatments.

**Table S4.** Values of metastasis-affected lung parenchyma area following intravenous CNT-based and/or chemotherapy treatments.

**Table S5.** Quantification of the *in vivo* effect of VRbp-CNT and Taxol® combination therapy.

**Materials and methods:**

**Analytical techniques:** LC-MS analyses were performed on a Thermo Fisher Finnigan LCQ Advantage Max system (EC 100/2 Nucleodur 100-3 C18ec column) integrated with a Thermo Scientific LCQ Fleet ion trap. HPLC analyses were performed on a Waters e2695 separations module instrument equipped with an autosampler and 2998 PDA detector, using a Nucleosil C18 column (150 × 4.6 mm), with a linear gradient of 0.1% TFA in water and 0.08% TFA in acetonitrile at a flow rate of 1.2 mL·min^-1^. Transmission electron microcopy (TEM) was performed on a Hitachi H7500 microscope (Tokyo, Japan) with an accelerating voltage of 80 kV, equipped with an AMT Hamamatsu camera (Tokyo, Japan). To prepare the TEM grids, the MWCNT conjugates were dispersed in water at 0.025 mg/mL by sonication. Ten microliters were deposited on carbon-coated copper TEM grids (Formvar/Carbon 300 Mesh, Cu from Delta Microscopies) and left for evaporation under ambient conditions. Thermogravimetric analysis (TGA) was performed using a Mettler Toledo TGA1 instrument using platinum pans with a ramp of 10°C·min^-1^ under N_2_ with a flow rate of 50 mL·min^-1^ from 100 to 900°C. X-ray photoelectron spectroscopy (XPS) was performed on a Thermo Scientific K-Alpha X-ray photoelectron spectrometer with a basic chamber pressure of 10^-8^ to 10^-9^ bar and an Al anode as the X-ray source (hν = 1486 eV). The samples were deposited on support via drop-casting on a silicon wafer coated with SiO_2._ The survey spectra are an average of 10 scans with a pass energy of 200 eV and a step size of 1 eV. An ion gun was turned on during the analyses. For each sample, the analysis was repeated three times. The Kaiser test was performed following the procedure described in (Kaiser, *et al.*, Anal Biochem. 34 (1970) 595–598). The dynamic light scattering (DLS) analyses were performed with a Zetasizer Nano ZS (Malvern, UK).

**Synthesis of Boc-amino PEG-Cy5 linker:** *N,N*-diisopropylethylamine (DIPEA) (30 µL), 1‑ethyl-3-(3-dimethylaminopropyl)carbodiimide (EDC) (12.4 mg) and *N*-hydroxysuccinimide (NHS) (6.6 mg) in anhydrous DMF (0.6 mL) under argon were added to a solution of cyanine 5 (Cy5) (25 mg) in anhydrous DMF (0.6 mL) at 4°C. The reaction mixture was stirred at room temperature for a period of 2 h, keeping it protected from light with an aluminum foil. Then, a solution of a commercially available Boc-amino PEG-amine (49 mg) in anhydrous DMF (2.3 mL) was added to the mixture, which was stirred for 1 day under argon at 4°C, keeping it protected from light. The day after, water (40 mL) was added, and the solution was lyophilized for 2 days. For purification, the product was dispersed in water (10 mL) and purified by a preparative HPLC system (Waters) on a Nucleosil C18 (1 x 30 cm) column (Macherey Nagel). The elution was achieved with a linear gradient of aqueous 0.1% TFA (A) and 0.08% TFA in acetonitrile (B) at a flow rate of 6 mL·min^-1^ with UV detection at 230 nm. The pure fractions were collected and lyophilized for 2 days (yield: 41%).

**Synthesis of NH_3_^+^-PEG-Cy5 linker:** TFA (2 mL) was added to a solution of Boc-amino PEG-Cy5 in MeOH (2 mL) previously sonicated in a water bath for 5 min. The mixture was stirred for 6 h at room temperature, keeping it protected from light. The reaction mixture was monitored by HPLC. TFA and MeOH were removed under reduced pressure, then water (30 mL) was added, and the sample was lyophilized for 2 days to yield NH_3_^+^-PEG-Cy5 (yield: 89%). The purity of the product was controlled by analytical RP-HPLC (Figure S7) and its molecular weight was assessed by LC/MS (Figure S8). MS (ESI, m/z): 1183 [M+H]^+^.

**Synthesis of ox-MWCNTs 1:** Pristine MWCNTs (80 mg) were dispersed in 12 mL of a mixture of sulfuric acid/nitric acid (3:1 v/v, 98%, and 65%, respectively). The reaction mixture was sonicated in a water bath for 24 h keeping the temperature below 35°C. The suspension was then carefully diluted with cold water (35 mL). The MWCNTs were filtered (membrane filtration, 0.45 µm), re-suspended in water, neutralized with an aqueous solution of NaOH 0.1 M, and filtered again. They were dispersed in water, and were dialyzed against water for 2 days and lyophilized.

The MWCNTs (40 mg) were dispersed in a solution of 1% SDS (40 mL). The suspension was sonicated in a water bath for 1 min and sonicated using a tip for 4 h. They were then filtered using a 0.1 µm Millipore membrane, washed with a solution of NaOH (0.1 M), and neutralized to reach a neutral pH. Finally, the suspension was lyophilized to yield the ox-MWCNTs **1** (74.5 mg).

**Synthesis of MWCNTs 2:** The ox-MWCNTs **1** (40 mg) were dispersed in oxalyl chloride (16 mL), sonicated in a water bath for 5 min, and heated at reflux for 24 h under argon. The oxalyl chloride was evaporated under reduced pressure and the nanotubes were dried under vacuum for 10 min. They were dispersed in a solution of Boc-TEG-NH_2_ (360 mg) in anhydrous DMF (25 mL) and the suspension was heated at reflux for 48 h under argon. The suspension was filtered (0.1 µm Millipore membrane), and the nanotubes were dispersed in DMF (50 mL), sonicated for 1 minute in a water bath, and filtered again. This process was repeated once with DMF (50 mL), twice with methanol (50 mL), and once with dichloromethane (50 mL). The CNTs were dispersed in water and dialyzed against water for 2 days to yield the functionalized MWCNTs **2** (38 mg) after drying under vacuum.

**Synthesis of MWCNT-NH_3_^+^ 3:** MWCNTs **2** (40 mg) were dispersed in 1,4-dioxane (20 mL). A solution of HCl 4 M in 1,4-dioxane (10 mL) was added. The suspension was sonicated in a water bath for 5 min and stirred for 6 h. The nanotubes were filtered (0.1 µm Millipore membrane), dispersed in DMF (50 mL), sonicated for 5 min in a water bath, and filtered again. This washing process was repeated twice with DMF, twice with methanol, and once with dichloromethane to yield MWCNT-NH_3_^+^ **3** (37 mg) after drying under vacuum.

**Synthesis of MWCNT-maleimide 4:** EDC (28.5 mg) and hydroxybenzotriazole (HOBt) (22.8 mg) were added to a solution of 6-maleimido hexanoic acid (35 mg) in anhydrous DMF (20 mL), which was previously sonicated in a water bath for 5 min. The reaction mixture was stirred at room temperature for 2 h under argon. A suspension of MWCNT-NH_3_^+^ **3** (25 mg) in anhydrous DMF (5 mL) and DIPEA (1 mL) under argon was added to the reaction mixture, which was stirred for 2 days under argon. The nanotubes were filtered (0.1 µm Millipore membrane), dispersed in DMF (50 mL), sonicated for 5 min in a water bath, and filtered again. This washing process was repeated twice with DMF and once with dichloromethane to yield the MWCNT-maleimide **4** (22 mg) after drying under a vacuum.

**Synthesis of MWCNTs 7:** To a suspension of ox-MWCNTs **1** (30 mg) in anhydrous DMF (25 mL) under argon, previously sonicated in a water bath for 5 min, EDC (9.8 mg) and HOBt (6.8 mg) were added. The mixture was sonicated in a water bath for 5 min and stirred for 2 h. A solution of the derivative NH_3_^+^-PEG-Cy5 (12 mg) and DIPEA (8 µL) in anhydrous DMF (5 mL) was added to the reaction mixture, which was sonicated for 5 min and then stirred for 2 days, keeping it protected from light. The nanotubes were filtered (0.1 µm Millipore membrane), dispersed in DMF (50 mL), sonicated for 1 min in a water bath, and filtered again. This process was repeated once with DMF (50 mL), twice with methanol (50 mL), and once with dichloromethane (50 mL). They were dispersed in water and dialyzed against water for 2 days to yield MWCNTs **7** (26 mg) after drying under vacuum.

**Synthesis of MWCNTs 8:** EDC (7 mg) and HOBt (5 mg) were added to a suspension of MWCNTs **7** (11 mg) in anhydrous DMF (10 mL), which was previously sonicated in a water bath for 5 minutes under argon. The mixture was sonicated in a water bath for 5 min and stirred for 2 h. A solution of the derivative Boc-TEG-NH_2_ (22 mg) in anhydrous DMF (5 mL) was added to the reaction mixture, which was sonicated for 5 min and stirred for 2 days, keeping it protected from light. The nanotubes were filtered (0.1 µm Millipore membrane), dispersed in DMF (50 mL), sonicated for 1 min in a water bath, and filtered again. This process was repeated once with DMF (50 mL), twice with methanol (50 mL), and once with dichloromethane (50 mL). The MWCNTs were dispersed in water and dialyzed against water for 2 days to yield MWCNTs **8** (10 mg) after drying under a vacuum.

**Synthesis of MWCNTs 9:** TFA (2 mL) was added to a suspension of MWCNTs **8** (10 mg) in methanol (2 mL), which was previously sonicated in a water bath for 5 min. The mixture was stirred for 6 h at room temperature, keeping it protected from light. The nanotubes were filtered (0.1 µm Millipore membrane), dispersed in methanol, sonicated for 1 min in a water bath, and filtered again. This process was repeated twice with methanol (50 mL) and once with dichloromethane (50 mL). The MWCNTs were dispersed in water and dialyzed against water for 2 days to yield MWCNTs **9** (9.6 mg) after drying under vacuum.

**Synthesis of MWCNTs 10:** EDC (7.3 mg) and HOBt (5.9 mg) were added to a solution of 6‑maleimidohexanoic acid (6.5 mg) in anhydrous DMF (2 mL) under argon. The mixture was sonicated in a water bath for 5 min and stirred for 2 h. A suspension of MWCNTs **9** (9 mg) and DIPEA (400 µL) in anhydrous DMF (3 mL) was added to the reaction mixture, which was sonicated for 5 min and stirred for 2 days. The nanotubes were filtered (0.1 µm Millipore membrane), dispersed in DMF (50 mL), sonicated for 1 min in a water bath, and filtered again. This process was repeated once with DMF (50 mL), twice with methanol (50 mL), and once with dichloromethane (50 mL). The MWCNTs were dispersed in water and dialyzed against water for 2 days to yield MWCNTs **10** (7 mg) after drying under a vacuum.

**Evaluation of the effect of ox-MWCNTs in angiogenesis *in vitro* and *in vivo*:** For the *in vitro* experiments, human umbilical endothelial cells (HUVEC, ATCC, CRL-1730) were cultured in endothelial cell medium supplemented with 10% fetal bovine serum (FBS), 1% endothelial cell growth supplement (ECGS), and 100 U/mL penicillin and 100 µg/mL streptomycin. The cells were maintained at 37°C in a 5% CO_2_ atmosphere. Blood vessel formation assays were conducted on Matrigel (Corning® Matrigel® Matrix). Then, cells were treated with 100 µg/mL of VRbp-CNT-Cy5, and the blood vessels were evaluated using phase contrast and fluorescence images. To quantify vasculature on *in vivo* experiments, representative confocal microscopy images of hematoxylin-eosin stained tumors from (i) untreated metastasis (control) and those treated with (ii) ox-MWCNTs, (iii) VRbp, and (iv) VRbp-CNT were analyzed by counting the number of erythrocytes *per* section.

**Effect of ox-MWCNTs on fibroblast, macrophages, and tumor cells**

Mouse fibroblasts NIH-3T3 (ATCC, CRL-1658) and mouse macrophage J774 (ATCC, TIB-67) were cultured in DMEM supplemented with 10% FBS and 100 U/mL gentamicin, at 37°C in 5% CO_2_. NIH-3T3, J774, and B16F10 melanoma cells were exposed to 100 µg/mL of ox-MWCNTs. After 96 h, cells were photographed with a phase contrast microscope, fixed with paraformaldehyde 4%, and stained with Hoechst dye. The evaluation was performed by flow cytometry using CytoFLEX equipment (Bectam Coulter), and data were analyzed using the CytExpert Software.

**Figure S1.** Synthesis of the peptide-CNT conjugates. For clarity, only one type of functional group is shown on the nanotubes.

We also prepared MWCNTs double functionalized with the same peptides and Cy5 to allow imaging of the conjugates *in vivo*. For this purpose, we designed a strategy based on the double amidation of ox-MWCNTs **1** (Figure S2). An amino-PEG-Cy5 linker (Cy5-PEG-NH_2_) was first coupled (using substoechiometric amount) to ox-MWCNTs **1** in the presence of coupling reagents allowing to functionalize only a part of the carboxylic acids, thus leaving part of COOH free for further peptide conjugation. This second step was performed using the same approach used for the preparation of the mono-functionalized MWCNTs. TGA confirmed the successive introduction of each functional group, as a gradual weight loss was observed after each step (Figure S11). TEM showed that the morphology of the fluorescent peptide-MWCNT conjugates was not affected by the different chemical treatments (Figures S5b-c), while fluorimetry data confirmed their strong fluorescence emission (Figure S12).

**Figure S2.** Synthesis of the fluorescent peptide-CNT conjugates **11** and **12**. For clarity, only one type of functional group is shown on the nanotubes.


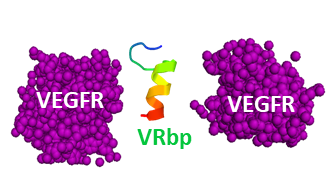


**Figure S3**. The 3D structure of the VEGF receptor-binding peptide (VRbp) was predicted and subsequently docked onto the VEGF receptor, as viewed from the top of the receptor homodimer. The structure of the VEGF receptor (reference: 5T89) was obtained from the PDB database. The peptide structure was predicted utilizing the PEP-FOLD 3D software.

**
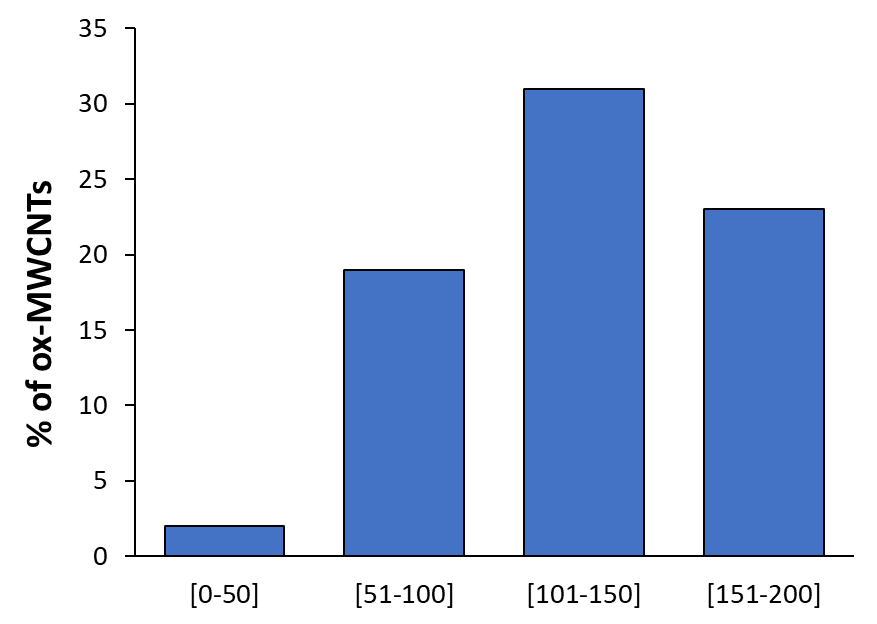
**

**Figure S4.** Length distribution of ox-MWCNTs **1**.


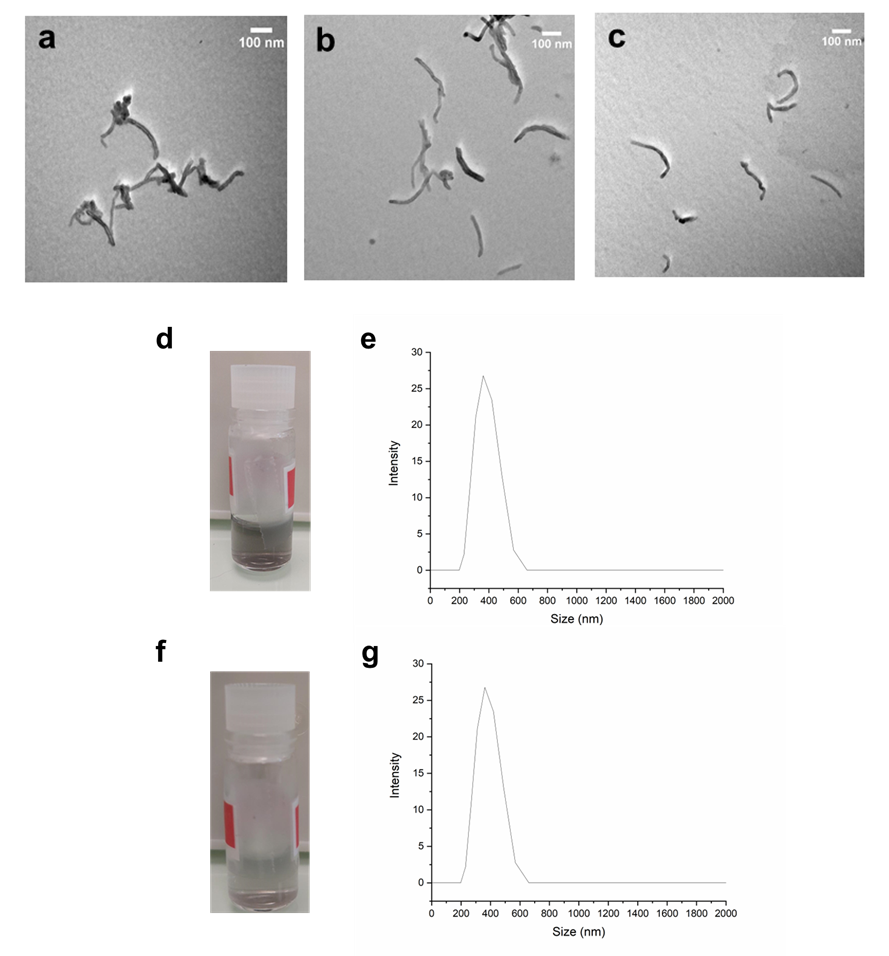


**Figure S5.** CNT characterization by TEM and DLS. TEM images of (a) CNT-VRbp **5**, (b) Cy5-CNT-VRbp **11** and (c) VRbp-CNT-Cy5 **12**. Photographs and hydrodynamic size distribution (assessed by DLS) of (d, e) CNT-VRbp **5** and (f, g) VRbp-CNT **6** dispersed in PBS.


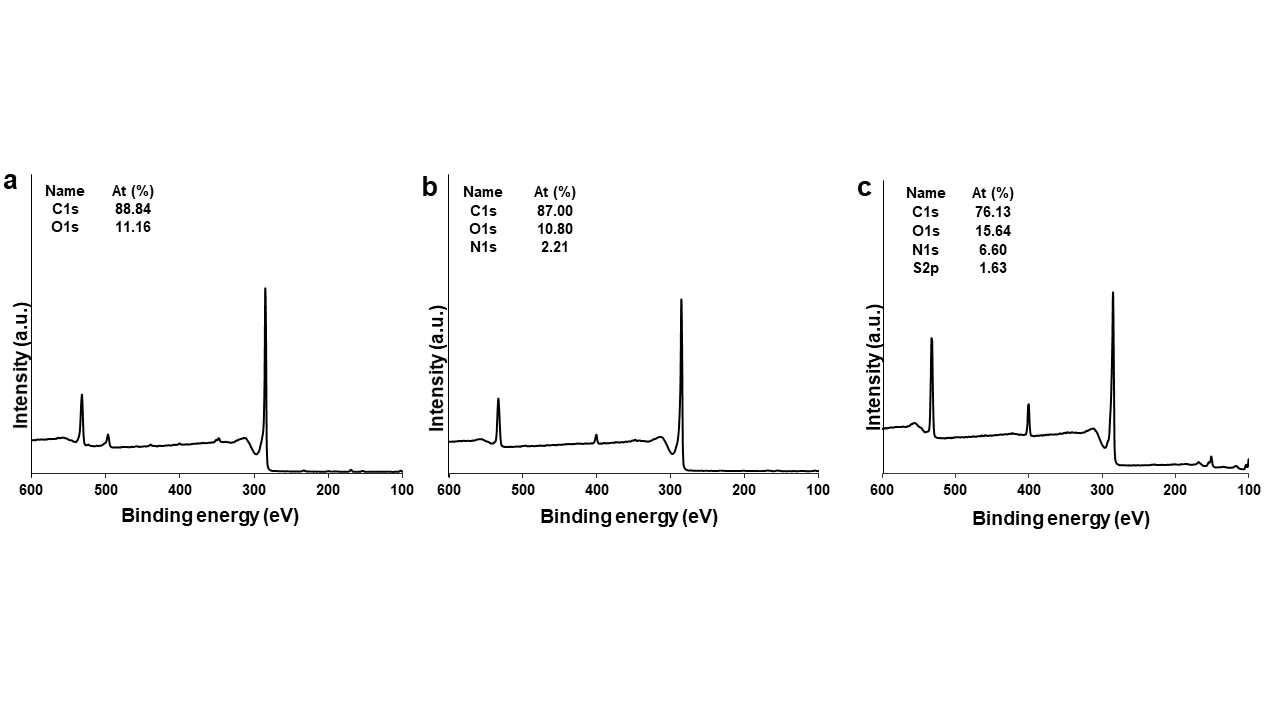


**Figure S6.** XPS survey spectra of (a) ox-MWCNTs **1**, (b) MWCNT-NH_3_^+^ **3**, and (c) CNT-VRbp **5**.


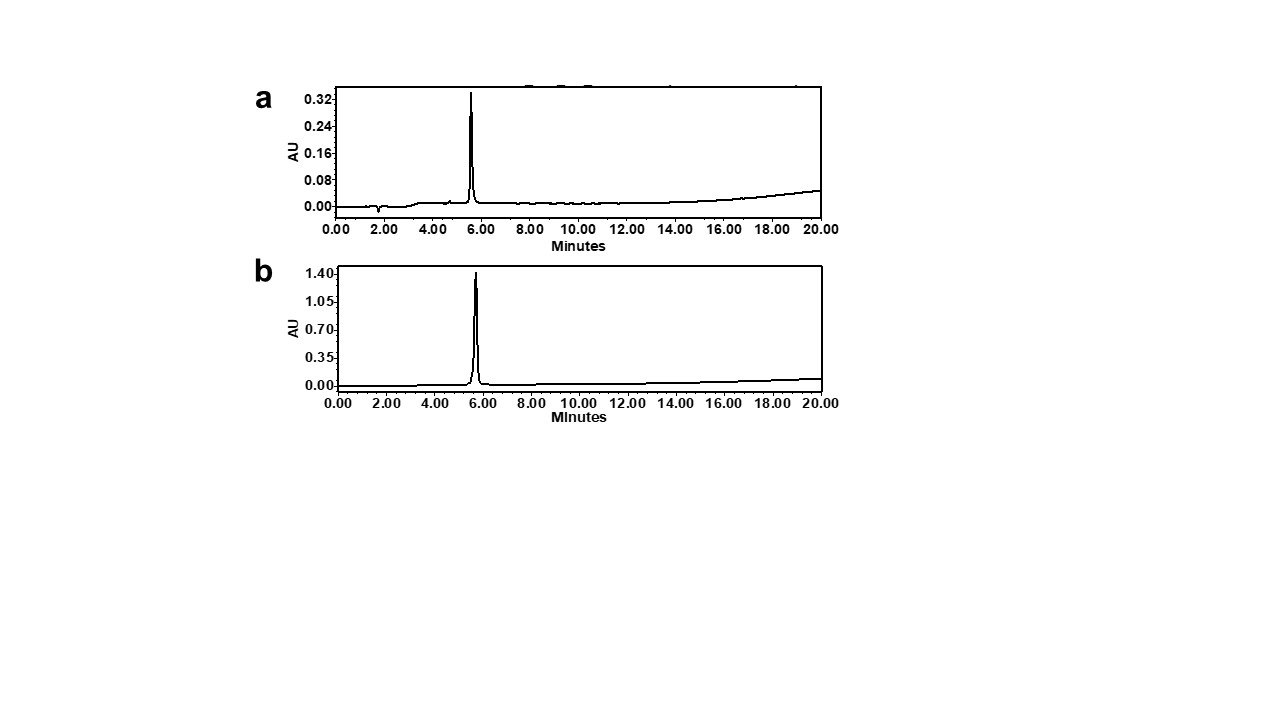


**Figure S7. HPLC chromatograms of (a) VRbp-N and (b) VRbp-C.**

**.**


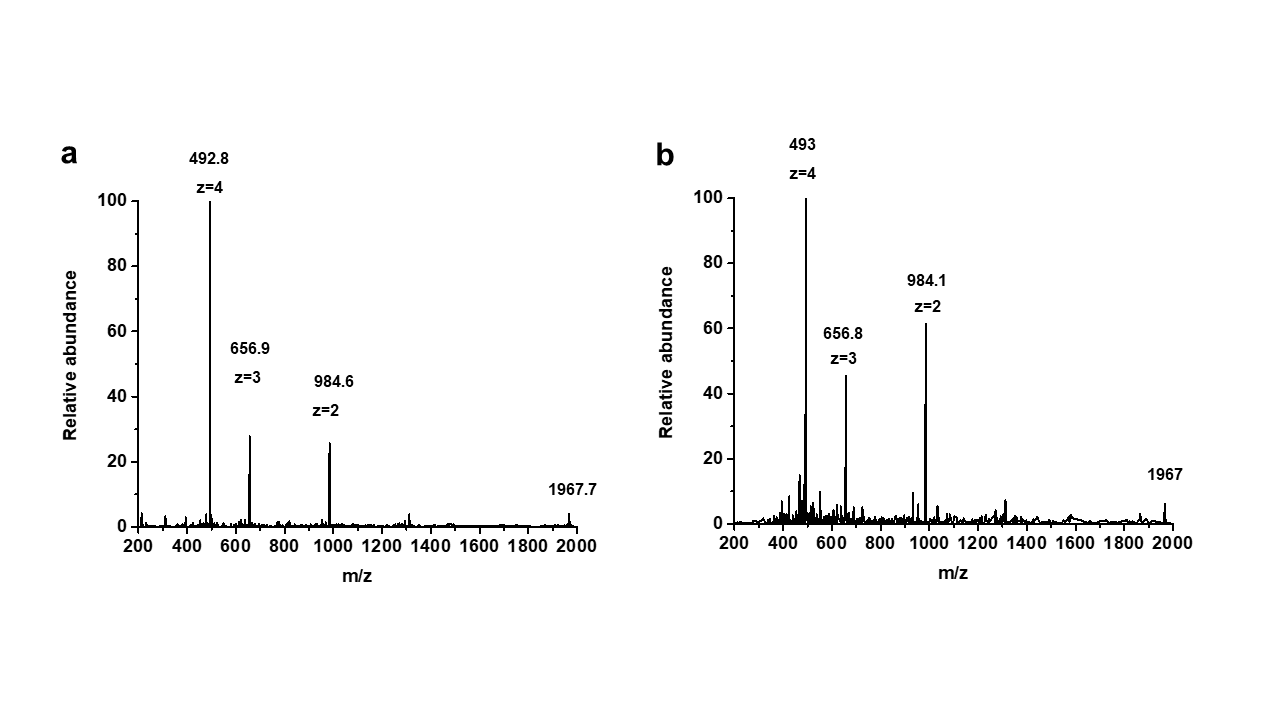


**Figure S8. Mass spectrometry data of (a) VRbp-N (**CGGGGGGHRHTKQRHTALH**) and (b) VRbp-C (**HRHTKQRHTALHGGGGGGC**).**


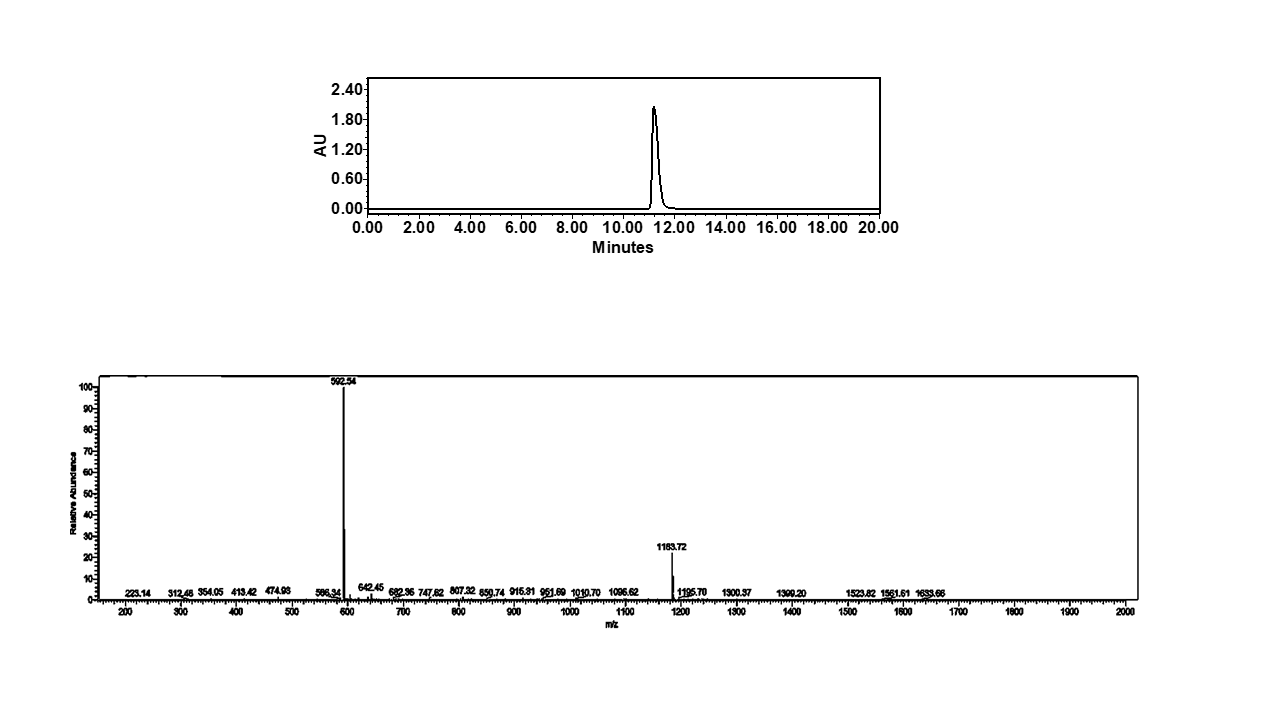


**Figure S9. HPLC of NH_3_^+^-PEG-Cy5 linker.**


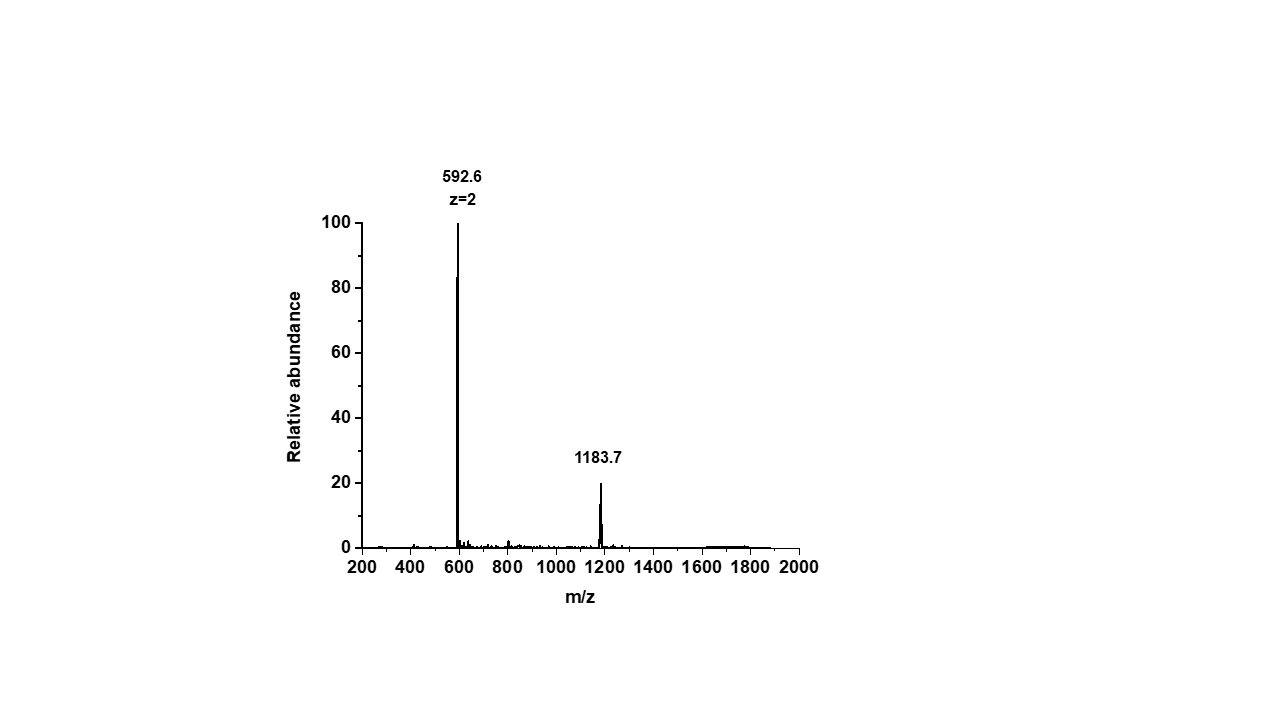


**Figure S10. Mass spectrometry of NH_3_^+^-PEG-Cy5 linker.**

**Figure S11.** TGA curves of the peptide-CNT-Cy5 conjugates and their precursors performed in N_2_ atmosphere.

**Figure S12.** Fluorescence spectra (λ_ex_: 620 nm) of the peptide-CNT conjugates **11** and **12**.

**
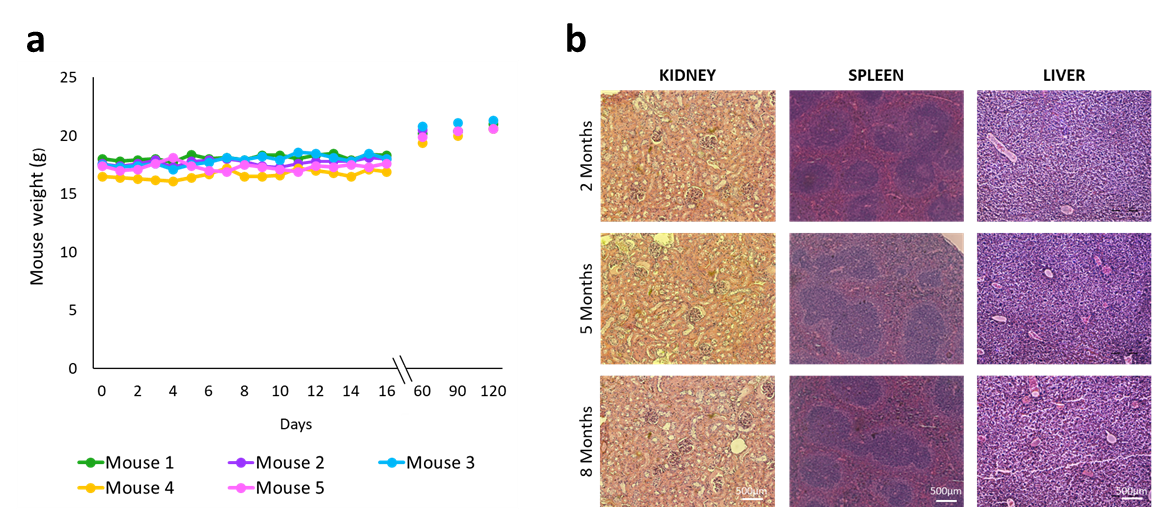
**

**Figure S13.** Toxicity assay. ox-MWCNTs showed no evident signs of toxicity. (a) Mice maintained their body weight after intravenous administration of 100 µg of ox-MWCNTs. (b) Histological sections of the liver, kidney, and spleen from animals at 2, 5, and 8 months post-injection, stained with hematoxylin-eosin, revealed no discernible alterations.


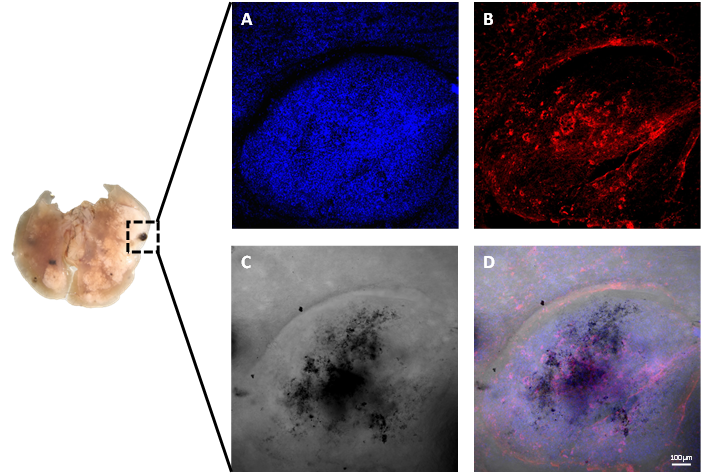


**Figure S14.** Representative images of metastatic lung tissue captured using both fluorescent confocal microscopy and phase contrast microscopy. (a) Nuclei are shown in the blue channel. (b) VEGFR in the metastasis tissue was immunostained with an anti-VEGF receptor antibody (red channel). Melanine (c) of the metastasis is visible in the phase contrast image. (d) Combination of the fluorescence and phase contrast images.


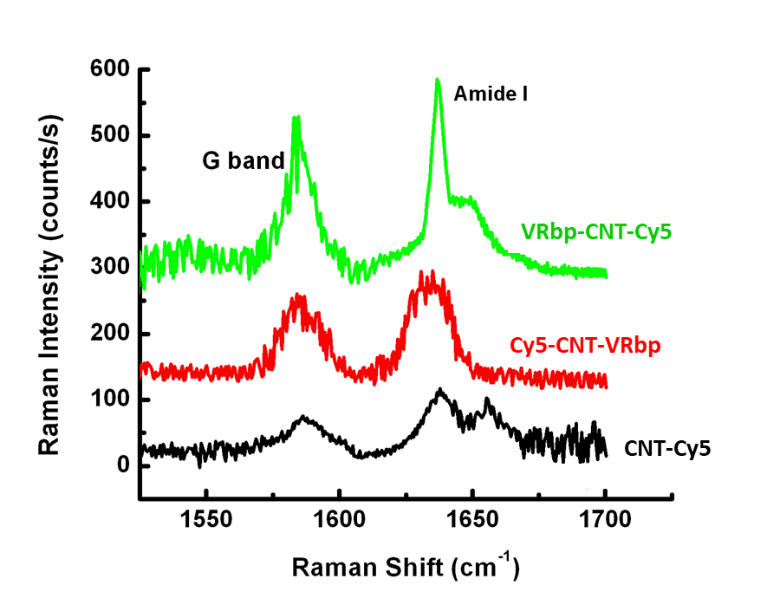


**Figure S15.** Raman spectra of lung tissue treated with CNT-Cy5 (black), Cy5-CNT-VRbp (red) or VRbp-CNT-Cy5 (green). The intensity of the G band and amide I band is much bigger in samples of lung tissues treated with Cy5-CNT-VRbp and VRbp-CNT-Cy5.


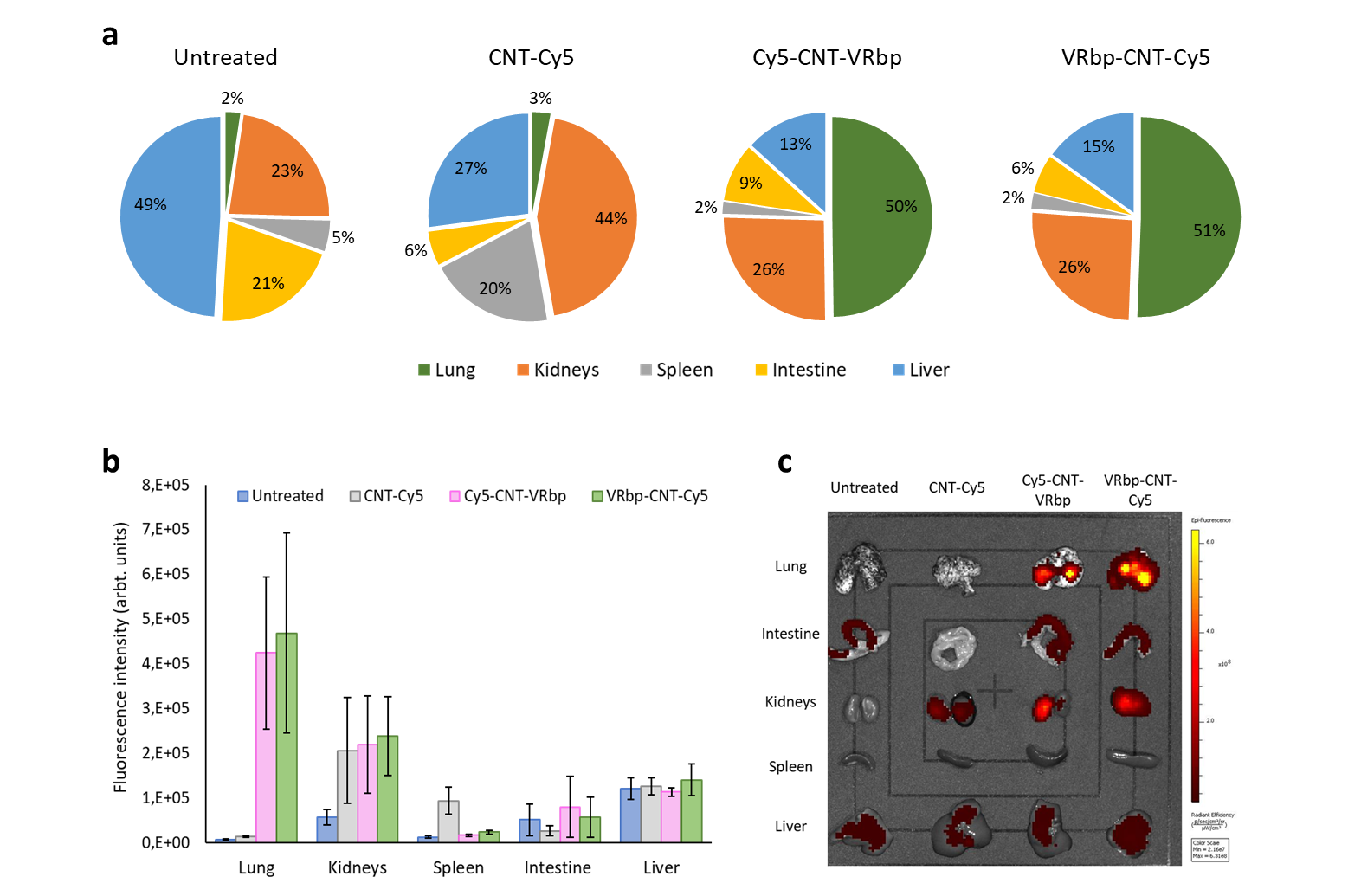


**Figure S16.** CNT targeting. (a) The percentage of CNT-Cy5, Cy5-CNT-VRbp, and VRbp-CNT-Cy5 in the main organs was quantified using IVIS® fluorescence imaging. (b) The fluorescence intensity was quantified at the time of sacrifice. Mean ± SD, *n* = 3. (c) *Ex vivo* fluorescence images of CNT-Cy5, Cy5-CNT-VRbp, and VRbp-CNT-Cy5 in isolated organs by IVIS® imaging.

*
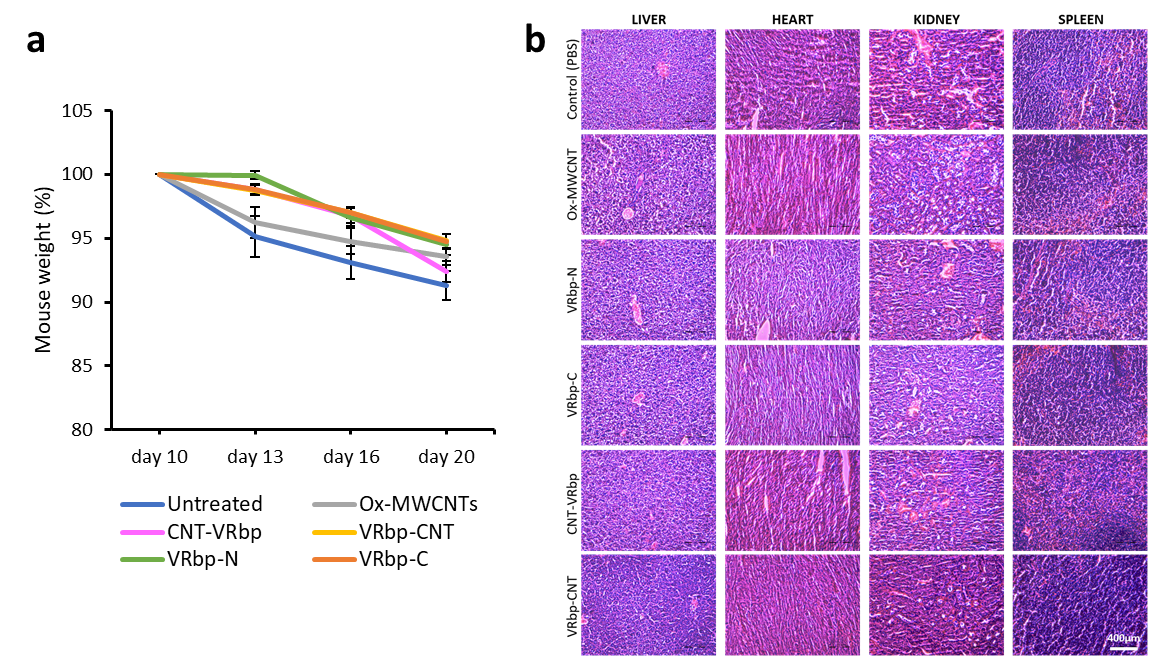
*

**Figure S17.** Animal monitoring studies. (a) Body weight monitoring of metastasis-bearing mice throughout the treatment. (b) Histological sections of the liver, heart, kidney, and spleen from the metastasis-bearing mice after receiving various treatments, stained with hematoxylin-eosin.


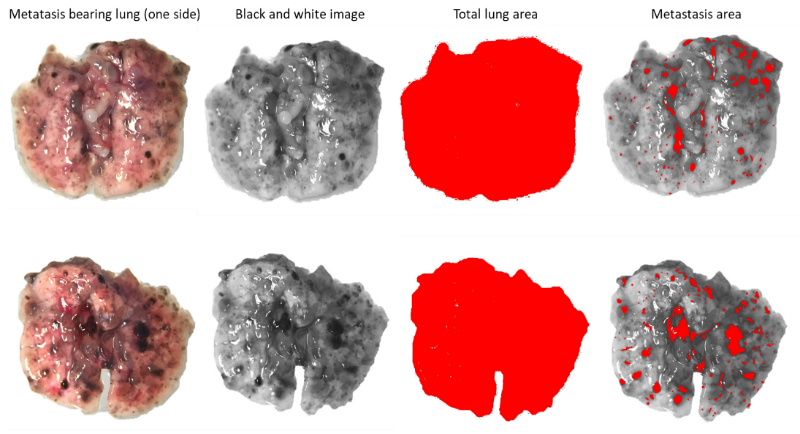

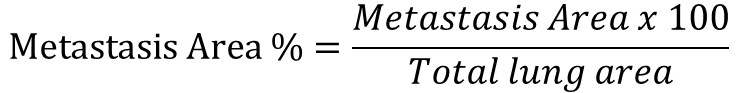


**Figure S18.** Lung metastasis quantification. The percentage of lung metastasis area was calculated considering the area of the metastatic foci versus the total area of the lung. Both the front and backside areas of the lungs were calculated separately.


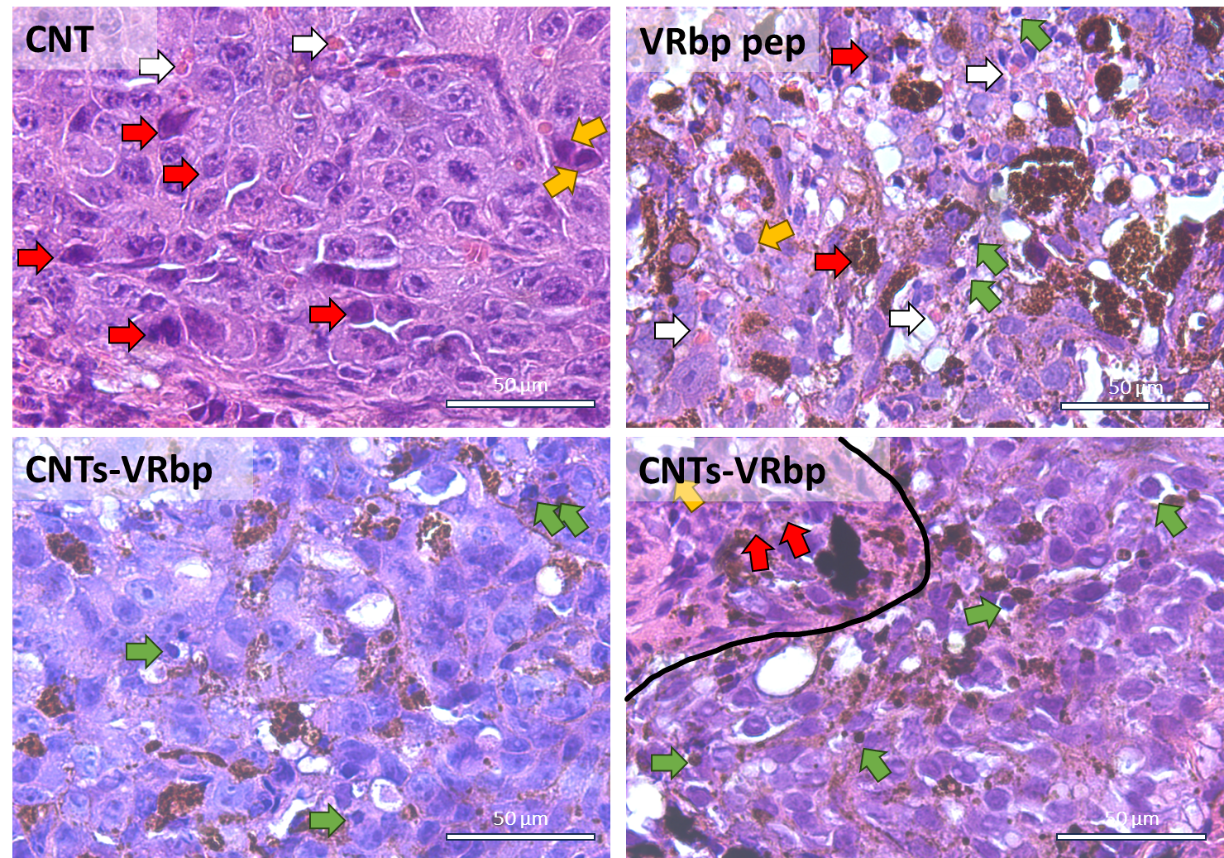


**Figure S19.** Micrographs of tumor paraffin sections stained with hematoxylin-eosin. A color-coded system, using arrows, aids in the morphological identification of various cell types. Macrophages are indicated by red arrows, while lymphocytes representing immune system cells are denoted by yellow arrows. Red blood cells, often surrounded by capillary cells and somewhat challenging to discern at these magnifications, are marked with white arrows. Additionally, apoptotic cell bodies are emphasized by green arrows. In the bottom-right image, a black line delineates the boundary between the micrometastasis and the surrounding capsule, where macrophage-like cells and lymphocytes can be observed.


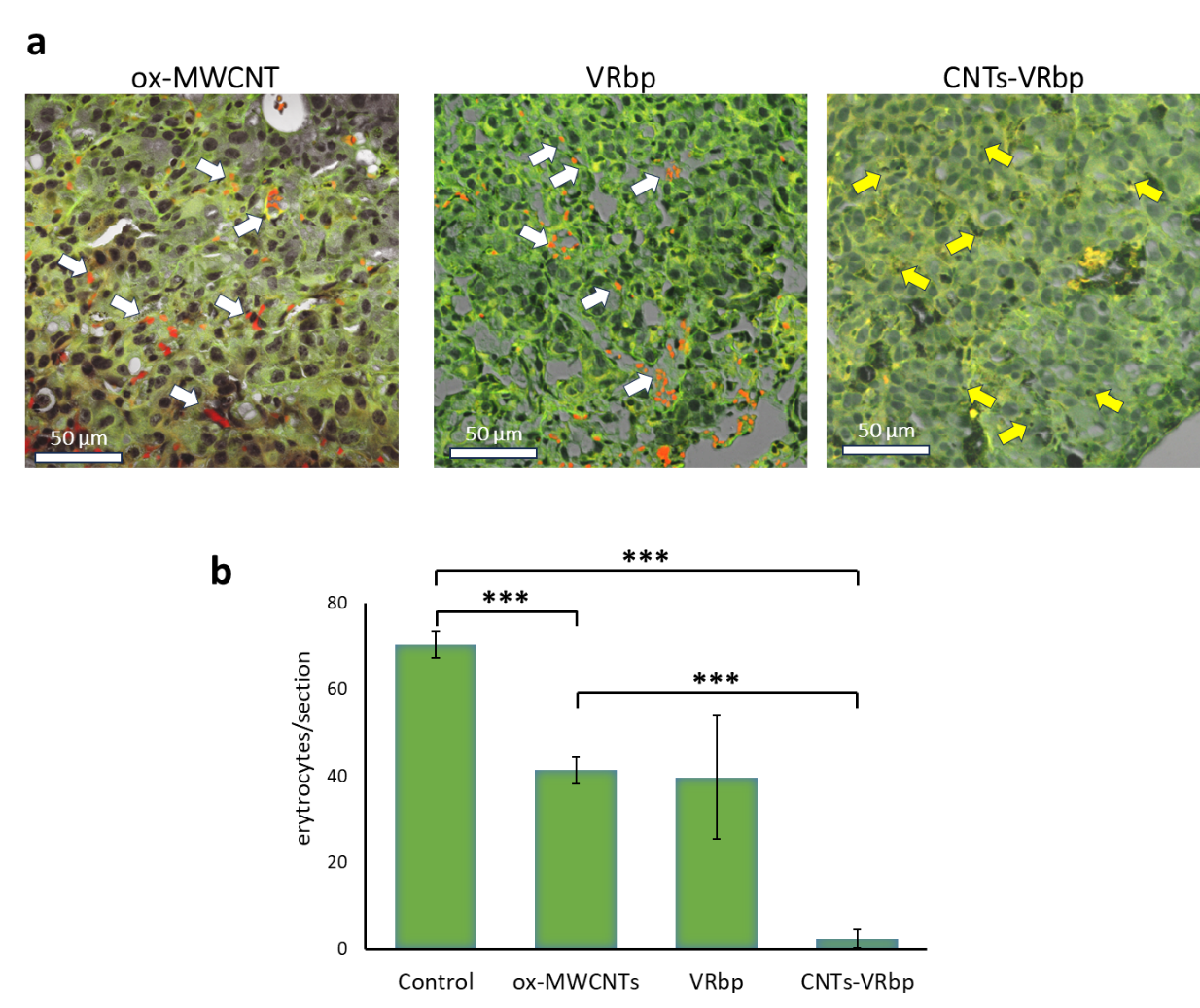


**Figure S20.** Intratumoral vascular study. (a) Confocal microscopy/phase contrast analysis of three representative lung metastases stained with hematoxylin-eosin in mice subjected to treatment with plain nanotubes (ox-MWCNTs), the peptide (VRbp), or the targeted nanotubes (CNT-VRbp). In the control groups treated with ox-MWCNTs or the peptide (indicated by white arrows), observable blood vessels containing red-stained erythrocytes are present. In contrast, the tumoral stroma in mice treated with VEGFR-targeted nanotubes displayed an absence of these discernible blood vessels. Additionally, an increased presence of apoptotic bodies was evident in the tumor sections treated with VRbp-CNT (highlighted by yellow arrows). (b) The quantification of red blood cells in tumoral tissue sections served as an indicator of metastasis vascularity. The statistical analysis revealed significant differences in the vasculature observed in sections from PBS-treated controls with ox-MWCNTs and with CNT-VRbp. The difference between ox-MWCNT and CNT-VRbp was also statistically significant (*n* = 3, *p* = 3. 1×10^-4^, 5.78×10^-6^, 5.27×10^-5^ comparing Control to ox-MWCNTs, Control to CNT-VRbp and ox-MWCNTs to VRbp-CNT, respectively; ****p* < 0.001).


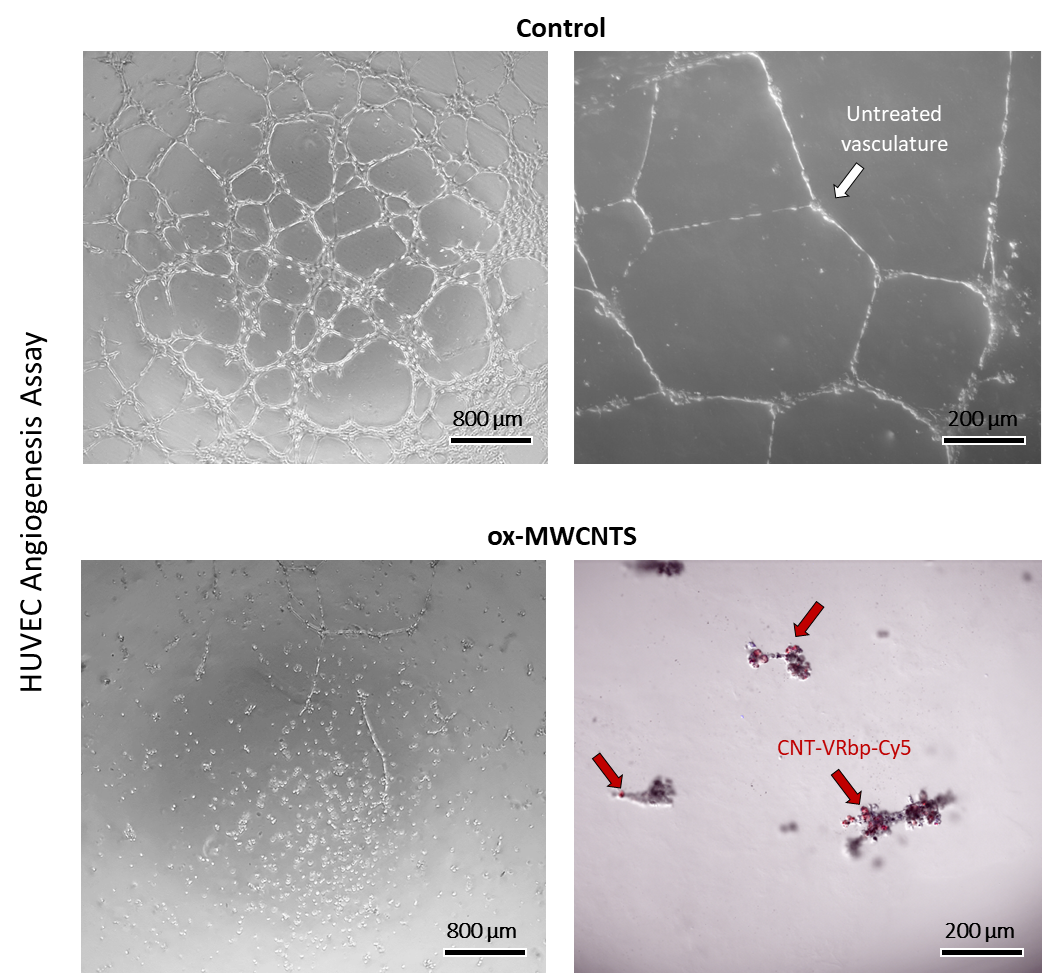


**Figure S21**. *In vitro* angiogenesis inhibition test. Assessment of angiogenesis tube formation with CNT-VRbp-Cy5. Representative images of HUVEC cells cultured for three days both, in the absence (top) and presence of CNT-VRbp-Cy5 (bottom). The untreated control cultures display a robust tubular vascular network (white arrow), in contrast to the cultures exposed to the CNT-VRbp-Cy5 where the vascular network is absent (bottom, right). The fluorescence emitted by the Cy5-labeled nanotubes (visible in the red channel) in the HUVEC cells is indicated by red arrows.


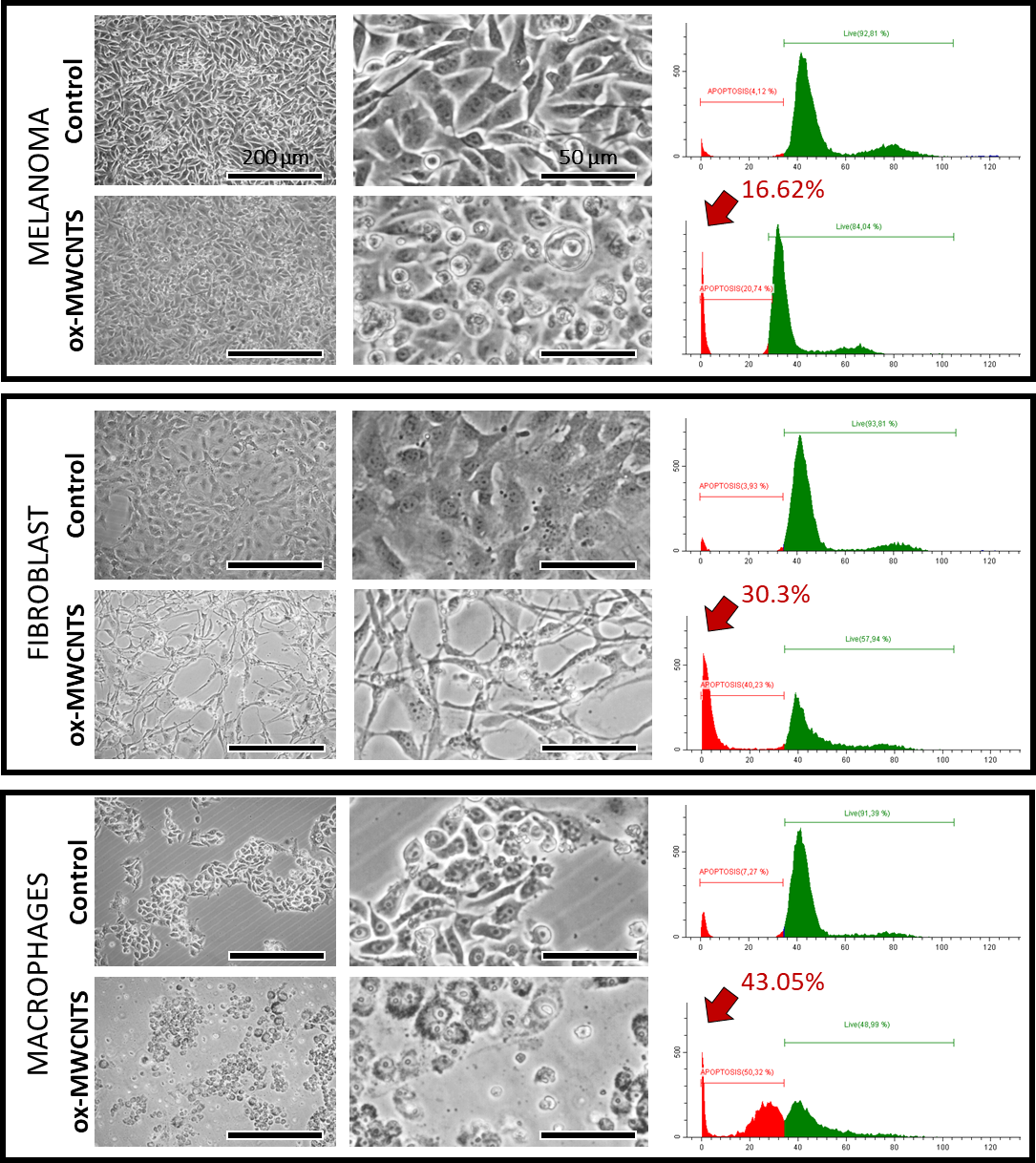


**Figure S22.** Phase-contrast images of cultures of murine malignant melanoma cells, NIH-3T3 murine fibroblasts, and BV2 murine macrophages treated or not with ox-MWCNTs (as indicated on the left). Cell death was documented by image analysis and quantified by flow cytometry in untreated controls or cultures exposed to ox-MWCNTs for 96 h. Live cells are shown in green. A marked rise in apoptosis (Sub-G_0_ fraction) is evident in cells treated with nanotubes (highlighted in red). The variance in the percentage of deceased cells between control and ox-MWCNT-treated cultures is visually depicted. It is worth noting that when exposed to identical conditions, melanoma cells are more resistant to the effects of ox-MWCNTs than fibroblasts and macrophages.

**Table S1. Biochemical blood parameters in treated animals (with ox-MWCNTs) and controls (Ctr).** The data shown represent the mean ± SD (*n* = 3).


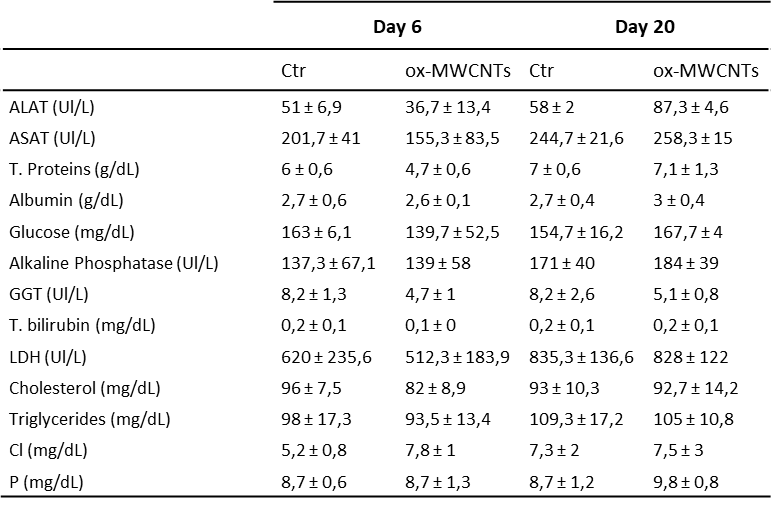


**Table S2. Hematological parameters in treated animals (with ox-MWCNTs) and controls (Ctr)**. The data shown represent the mean ± SD (*n* = 3).


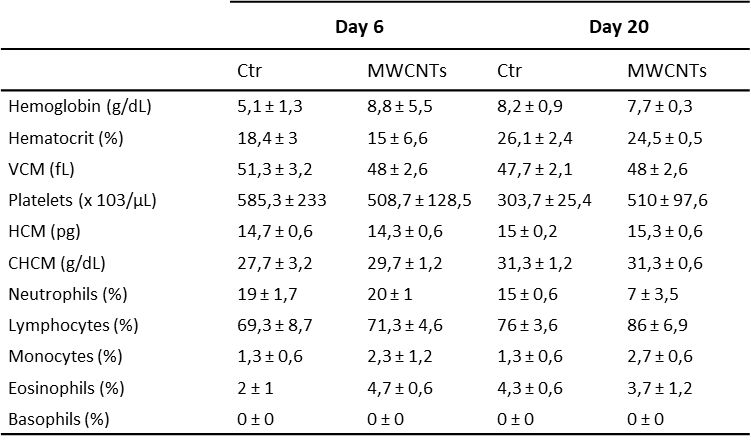


**Table S3. Values of metastasis-affected lung parenchyma area following different intravenous treatments.**


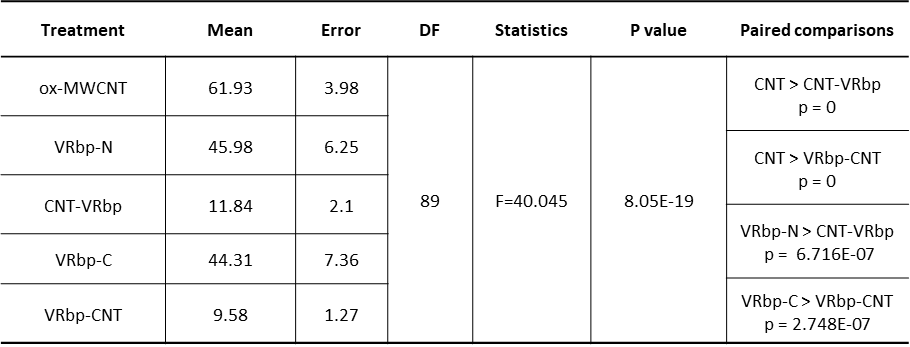


**Table S4. Values of metastasis-affected lung parenchyma area following intravenous CNT-based and/or chemotherapy treatments.**


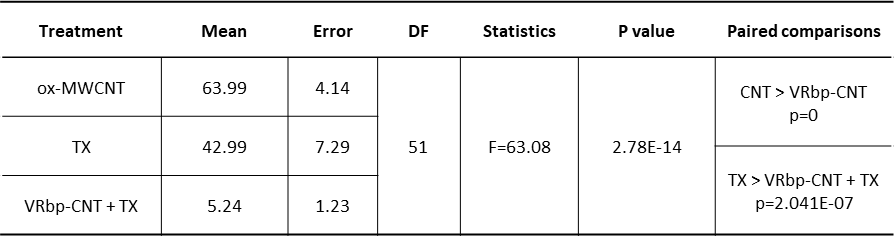


**Table S5. Quantification of the *in vivo* effect of VRbp-CNT and Taxol® combination therapy.**

| Effect | Formula* |
| --- | --- |
| Synergistic | $\left( A+B \right)< \frac{A\times B}{100}$ |
| Additive | $\left( A+B \right)= \frac{A\times B}{100}$ |
| Sub-additive | $\frac{A\times B}{100}<\left( A+B \right)<A;if A<B$ |
| Interference | $A<\left( A+B \right)<B, if A<B$ |
| Antagonistic | $B<\left( A+B \right), if A<B$ |

In this case: A = VRbp-CNT = 11.8%

B = TX = 44.6%

(A+B) = VRbp-CNT+TX = 5.24%

According to the formulas above, the effect of the combination of VRbp-CNT and Taxol® is **additive**: (A×B)/100=5.26% = (A+B)=5.24%
